# Differential Activation of Wnt-β-Catenin Pathway in Triple Negative Breast Cancer Increases MMP7 in a PTEN Dependent Manner

**DOI:** 10.1371/journal.pone.0077425

**Published:** 2013-10-15

**Authors:** Nandini Dey, Brandon Young, Mark Abramovitz, Mark Bouzyk, Benjamin Barwick, Pradip De, Brian Leyland-Jones

**Affiliations:** 1 Edith Sanford Breast Cancer, Sanford Research, Sioux Falls, South Dakota, United States of America; 2 VM Institute of Research, Montreal, Quebec, Canada; 3 Human Genetics, Emory University, Atlanta, Georgia, United States of America; 4 Winship Cancer Institute, Emory University School of Medicine, Atlanta, Georgia, United States of America; 5 The Scripps Research Institute Jupiter, Florida, United States of America; 6 Internal Medicine, University of South Dakota, Sioux Falls, South Dakota, United States of America; King's College London, United Kingdom

## Abstract

Mutations of genes in tumor cells of Triple Negative subset of Breast Cancer (TNBC) deregulate pathways of signal transduction. The loss of tumor suppressor gene PTEN is the most common first event associated with basal-like subtype (Martins, De, Almendro, Gonen, and Park, 2012). Here we report for the first time that the functional upregulation of secreted-MMP7, a transcriptional target of Wnt-β-catenin signature pathway in TNBC is associated to the loss of PTEN. We identified differential expression of mRNAs in several key-components genes, and transcriptional target genes of the Wnt-β-catenin pathway (WP), including *beta-catenin, FZD7*, *DVL1, MMP7*, c-*MYC*, *BIRC5*, *CD44*, *PPARD*, c-*MET, and NOTCH1* in FFPE tumors samples from TNBC patients of two independent cohorts. A similar differential upregulation of mRNA/protein for beta-catenin, the functional readout of WP, and for MMP7, a transcriptional target gene of beta-catenin was observed in TNBC cell line models. Genetic or pharmacological attenuation of beta-catenin by SiRNA or WP modulators (XAV939 and sulindac sulfide) and pharmacological mimicking of PTEN following LY294002 treatment downregulated MMP7 levels as well as enzymatic function of the secreted MMP7 in MMP7 positive PTEN-null TNBC cells. Patient data revealed that MMP7 mRNA was high in only a subpopulation of TNBC, and this subpopulation was characterized by a concurrent low expression of PTEN mRNA. In cell lines, a high expression of casein-zymograph-positive MMP7 was distinguished by an absence of functional PTEN. A similar inverse relationship between MMP7 and PTEN mRNA levels was observed in the PAM50 data set (a correlation coefficient of -0.54). The PAM50 subtype and outcome data revealed that the high MMP7 group had low pCR (25%) and High Rd (74%) in clinical stage T3 pathologic response in contrast to the high pCR (40%) and low residual disease (RD) (60%) of the low MMP7 group.

## Introduction

Triple negative (TN; negative for hormone receptors, And HER2 amplification/overexpression) and/or basal-like subtypes of breast cancer (BC) present the most challenging diagnosis among BC patients as it confers a poor clinical outcome due to (1) a diverse group of multiple genetic alterations in the refractory RD, (2) a frequent and an aggressive nature of metastasis, and (3) a limited number of available targeted therapeutic options owing to a poorly understood biology [1–3]. Despite recent reports that indicated the involvement of certain genes/signaling molecules related to tumorigenic pathways [4–8] in TNBC (includes both TNBC and basal-like hereof), there remains an unmet need for an in-depth study to identify major genetic events associated to driver pathways in this subtype of BC. Recently, Kornelia Polyak’s team [9] demonstrated the importance of PTEN loss in the relative temporal order of somatic events associated with basal-like subtype to show that PTEN loss is the most common first event associated with basal-like subtype.

The WP is a ligand-driven signaling pathway that regulates several cellular phenotypes in the development, and the progression of cancers, including breast [10–12]. Earlier studies by Jonsson et al., on the involvement of APC/beta-catenin signaling in human breast cancer indicated that it is imperative to identify elements that selectively drive the oncogenic activity of beta-catenin in breast cancer [13]. Although WP deregulation (via expression of Wnt-ligands, or secreted Wnt antagonists or APC inactivation) have been observed in BC [14], the mechanism of WP-activation in different BC subtypes as well as its association with the clinical outcome in BC still remains either rudimentary or a matter of controversy since confirmatory reports relating to the mechanism of WP’s involvement in breast tumorigenesis had been mostly obtained from mouse models [15].

Following the pioneering work of Perou *et al*. [16], several studies have demonstrated a consistent correlation between genetic profile of tumor subtypes and their clinical outcomes [17,18]. Thus, it is important to appreciate the subtype-specific involvement of the WP-activation in TNBC, its relationship to the most common first genetic event in TNBC, and its relevance in the context of patient outcome in TNBC. The extent of WP’s involvement in the genesis, and the progression of the TN subtype of BC begun to unfold from the works of Reis-Filho’s team [15] and ours [19,20] (*MS resubmitted*). Although WP’s involvement in the subtype specific BC has been reported recently, in the context of HMGA2 induction and proliferation in metastatic triple-negative breast cancer [21], its role in the context of BC heterogeneity, subtype specific genomic events, and outcome remains undecided.

Here we have demonstrated that (1) there is a subtype specific upregulation of the WP-activation in TNBC as compared to the luminal (HR+) or HER2+ tumors, and (2) WP-mediated high expression of mRNA of MMP7, a transcriptional target of beta-catenin, is associated with the functional loss of PTEN gene, the most common first event associated with TNBC subtype [9]. Our data also demonstrate that MMP7 is differentially upregulated in a subset of TNBC characterized by the lower PTEN mRNA. The PAM50 outcome data, wherein a similar inverse relationship between MMP7 and PTEN mRNA levels was observed (a correlation coefficient of -0.54), revealed that the patients in high MMP7 group had low pCR (25%) along with High Rd (74%) in clinical stage T3 pathologic response in contrast to the high pCR (40%) and low RD (60%) of the low MMP7 group.

## Materials and methods

### Patient samples and Study cohorts

Samples (archived formalin-fixed, paraffin embedded (FFPE) tumor specimens from St. Mary’s Hospital Centre in Montreal, Canada; Montreal cohort) were analyzed at the Emory Biomarker Service Center (Emory University) (*Data deposition: Microarray data curetted under GEO series accession numbers* [*GSE17650 & GSE18539*]). FFPE breast tumor paraffin blocks from women with confirmed invasive carcinomas of the breast were handled according to standard tissue acquisition protocols. We obtained FFPE samples of breast carcinomas that had previously been scored for the BC biomarkers, ER, PR and HER2 by immunohistochemistry (IHC). In some IHC equivocal cases of HER2-staining (IHC two+), fluorescent in Situ hybridization (FISH) was used to confirm genomic amplification as described earlier [22]. The specific samples used in this study have been described in previous publications [19,22,23].

### RNA preparation, quality control, quantitative RT-PCR and DASL assay

Total RNA was prepared from three × 5 µm FFPE sections. RNA de-paraffinization, extraction, and purification were performed using a commercially available RNA High Pure Kit (Roche, Mannheim, Germany) modified as previously described [22–24]. Samples, including technical replicates (singleton to quadruplicate), were run in the DASL (cDNA-mediated Annealing, Selection, extension, and Ligation) assay on two universal array matrices (UAMs). The hybridized UAMs were scanned using the BeadStation 500 Instrument (Illumina Inc.).

### Illumina direct-Hyb assay & RT-PCR

The extracted RNA was quantified on a NanoDrop (NanoDrop Technologies Inc., Wilmington, DE) and normalized for analysis on the Illumina Direct-Hyb gene expression assay on Illumina Human HT-12 beadchips following the manufacturer’s instructions as described earlier [22]. Expression of specific transcripts was further confirmed by quantitative RT-PCR (each sample performed in triplicate) on an Applied Biosystems ABI 7900, and the following commercially available TaqMan primers were used: Hs01042796_m1 (MMP7), Hs01053430_m1 (BTRC), Hs01062006_m1 (NOTCH1), Hs00275833_s1 (FZD7), and Hs00737028_m1 (DVL1) [25].

### Cell lines, reagents and antibodies

Cell line data were downloaded from GEO (GD3285) with replicates averaged for each cell line. All breast tumor cell lines, except SUM102, SUM149 (Asterand; Partners in human tissue research) and Herceptin (trastuzumab) resistant BT474HR (BTH) (obtained from Dr. Mark Pegram; Div. Hemat. & Med. Oncology, UCLA, CA) cell lines, were obtained from ATCC. DKAT cells are kindly provided by Dr. Victoria L. Seewaldt, Department of Medicine, Duke University Medical Center, Durham, North Carolina). Antibodies against c-Myc, c-Met, cyclin D1, PCNA, PTEN, pAKT, AKT (Cell Signaling Technology, MA), tubulin (BD Biosciences, CA), TCF-4 (Upstate, Millipore CA), DVL-1, axin (Santa Cruz Biotechnology, CA), survivin (Alpha Diagnostic Intl. Inc., TX), beta-catenin, MMP2, MMP7, MMP9, and MMP14 (Abcam Inc., Cambridge, MA) were used.

### Cell culture, treatments and biochemical analysis

Breast tumor cell lines included TN (Figure S3), HR+ (MCF-7, CAMA1, T47D, ZR-75-1), and HER2+ (BT474, BTH474, HCC1954 and SKBr3) and were all cultured according to standard protocols. Normalized lysates (20-40-µg protein) were resolved by 10% SDS-PAGE for Western blot analysis [26,27]. Cell lines were treated with XAV939 (WP-modulator that stabilizes axin) [28,29], sulindac sulfide (attenuates cellular levels of beta-catenin) [30], and pan PI3K inhibitor (LY294002).

### Caesin zymography

Enzymatic activity of the secreted MMP7 from the conditioned-media was determined by zymography using Bio-Rad precast caesin gels [24,31].

### Silencing of Beta-catenin and PTEN in TNBC cells

Cell lines were transiently transfected with human-specific beta-catenin SiRNA (Invitrogen Inc.) and PTEN SiRNA (SMARTpool, SiGEMONE; Thermo, Fisher Scientific) using Lipofectamine 2000 (Invitrogen Inc.) as described earlier [26]. In brief, cells were grown in 6 well tissue culture plates to 60–70% confluency and then transfected with SiRNA plasmids using Lipofectamine. Cells were harvested at 72 hours. Beta-catenin, PTEN, pGSK3β, GSK-3β and MMP7 protein levels were determination of by Western blot [32].

### Migration and invasion of TNBC cells

Fibronectin-directed migration (widths of the scratch as measured in Metamorph; AxioVision), fibronectin-directed invasion (basement membrane coated-transwell, BD Pharmingen) were performed as described earlier [33].

### TCF/LEF promoter activity assay

A luciferase-based reporter gene was used to measure promoter activity of the TCF/LEF transcription factor [34]. In the TCF/LEF promoter assay experiments, cells were co-transfected either with TOP Flash or FOP Flash using Lipofectamine and then treated with sulindac sulfide. In brief, cells were co-transfected with 2.5-µg top flash, a synthetic luciferase-based promoter plasmid (sensitive to the activity of the β-catenin/ TCF-4 complex, containing 3 copies of the TCF-4 binding site upstream of a firefly luciferase reporter gene) using the Lipofectamine 2000. In the other set of cells, an equal amount of the mutant form of the above promoter (FOP Flash) were co-transfected using the same transfection reagent. After 12-h incubation, each set was treated with sulindac sulfide for 24 hours. The relative luciferase activity (TOP Flash/FOP Flash) was calculated from triplicate experiments.

### Statistical analysis

Data were analyzed for hierarchical clustering using the heatmap.2 function of the g-plots package in R bioconductor where the Euclidean distance dissimilarity metric was used in combination with an average clustering algorithm. Bar plots were created in Excel with error-bars denoting 1 standard error and significance was determined by unpaired t-tests for unequal sample sizes and means with a p-value < 0.05. DASL transcript intensities were interpreted in GenomeStudio. Samples with insufficient signal-to-noise ratios (< 3) were removed from subsequent analysis and the remaining samples were quantile normalized with plate scaling. Technical replicates within samples were average combined to create one signature per tumor.

## Results

### Differential upregulation of mRNAs for the WP components and transcriptional targets of WP in tumors from TNBC patients

**Figure 1:**
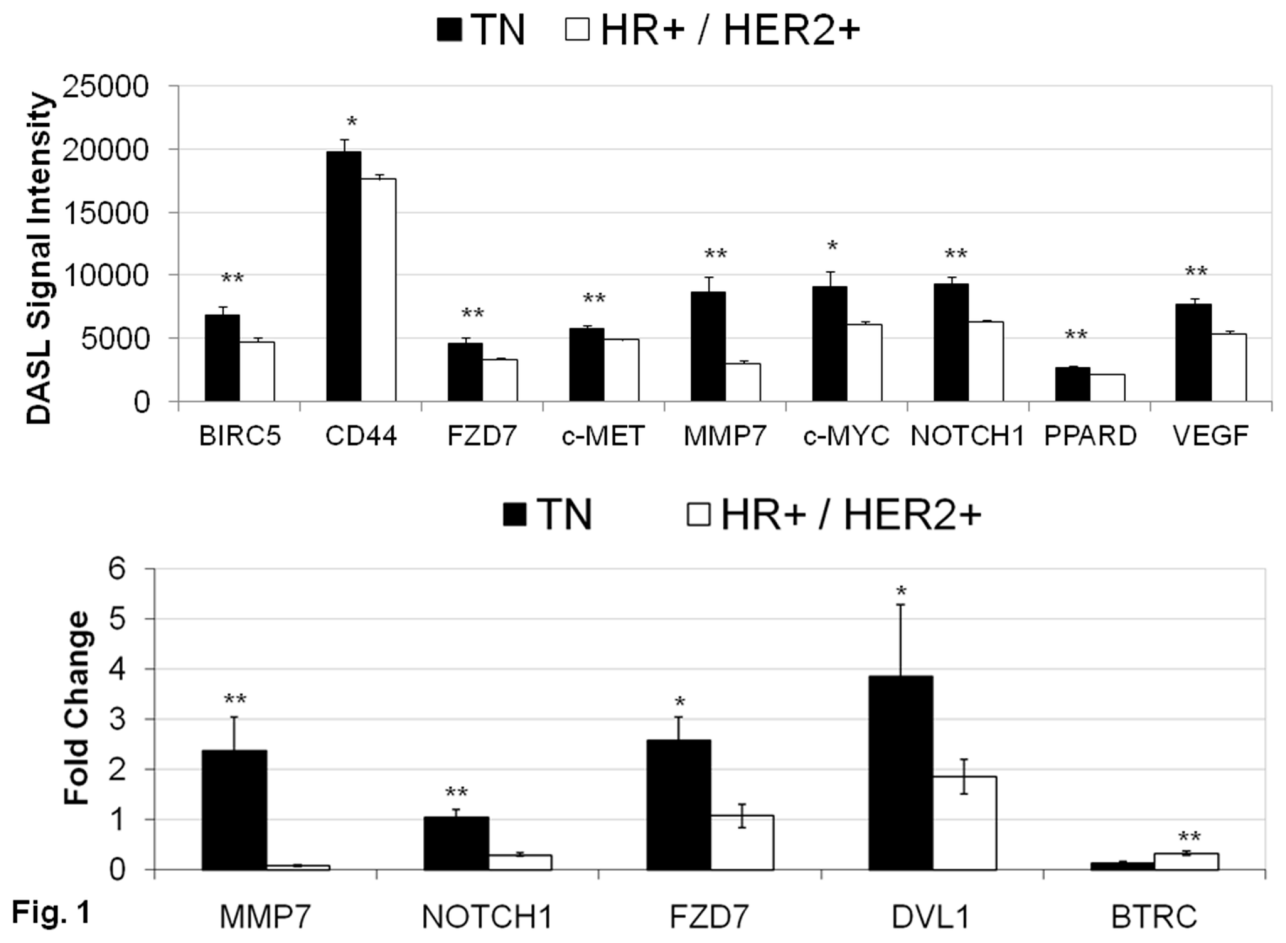
Expression of mRNA for a number of different components of Wnt-β-catenin pathway (WP) and transcriptional targets of WP in Triple Negative subset of Breast Cancer (TNBC) patients. Figure shows the expression of mRNAs for different components of Wnt-signaling in the Montreal cohort (upper panel). Average DASL signal intensity of Wnt transcriptional targets in Triple Negative (TN) tumors (black) are compared to non-TN (HR+ and HER2+) tumors (white). p-values < 0.05 determined using a t-test for unequal variances and Bonferroni’s correction for 46 Wnt related genes on a custom breast cancer DASL panel used to study the expression profile of the Montreal cohort (upper panel). RT-PCR confirmation of Wnt-signaling components in the Montreal cohort is shown (lower panel). Wnt transcriptional targets were validated using RT-PCR (10 TN and 10 HR+ patients in the Montreal cohort). The Wnt transducer DVL1 and the Wnt agonist BTRC were analyzed by RT-PCR and both increased (indicative of an increased Wnt signaling) in TN tumors. Asterisks (*) indicate p-values < 0.01 of differential regulation using two-sided t-tests for unequal variances and Bonferroni’s correction for multiple hypothesis (lower panel).

We observed that WP transcriptional targets *MMP7*, c-*MYC*, *BIRC5*, *CD44*, *PPARD*, and c-*MET*, were differentially upregulated in TN tumors as compared to the non-triple negative (HR+ and HER2+) tumors. Additionally, mRNA for the Wnt-ligand receptor *FZD7*, and the downstream target *NOTCH1* were also upregulated in the same set of patients (Fig. 1). The mRNA levels for several critical components of the pathway (*BIRC5, CD44, FZD7, c-MET, MMP7, c-MYC, NOTCH1, PPARD*, and *VEGF*) were significantly increased (DASL signal intensity) in TN tumor samples as compared to non-TN tumor samples (upper panel). To validate the DASL observations, we performed quantitative RT-PCR on the representative upregulated genes, and observed that the expression of mRNAs, including *MMP7, NOTCH1, FZD7*, were significantly higher in TN tumor samples than in non-TN tumor samples (lower panel). We also observed a fold increase in the expression of DVL1 (and BTRC to a lesser extent) in TN tumor samples as compared to non-TN tumor samples.

### Differential upregulation of WP components and transcriptional targets of WP in cell line-based model of TNBC

We examined mRNA expression of several key components and transcriptional target proteins of the WP in HCC38, MDA-MB468, and HCC70 TN breast cancer cell lines, and showed that expression of *AXIN, DVL1, and TCF4* were significantly increased in TN breast cancer cell lines as compared to the non-TN breast cancer cell line, MCF7 (Figure S5). Although expression of *MYC* varied between the three TN cell lines, their average expression was higher than that in MCF7 cells. We examined several Wnt ligands, including *WNT3A, WNT5A, WNT5B, WNT6, WNT7B, WNT9B, and WNT11* (Figure S5). The modest increase in mRNA expression was found to be statistically significant for *WNT7B* and *WNT9B*. An overall trend was noted towards an increased expression of other ligands in the TN cell lines as compared to the MCF7 cell line. A similar trend was observed for the mRNA expression for both *FZD4* receptor and *LRP6* co-receptor. We also observed an increase in the expression of additional target genes of the pathway, namely, *c-Jun, VEGFA* and *PLAUR* in TN breast cancer cell lines. The expression of *AXIN*, *DVL1*, and *TCF4* showed variable levels in different TN breast cancer cell lines.

Our data (Figure S6) shows the level of protein-expression of several transcriptional targets of the WP in different BC cell lines (PCNA was used as an internal standard). c-MYC protein was high in most TN breast cancer cell lines, including MDA-MB231 and MDA-MB468 as were levels of cyclin D1 in several TN breast cancer cell lines, including HCC38 and HCC1937. With the exception of SKBr3 cells (lane 3) that expressed a high level of c-Met protein, many TN cell lines, including HCC38 and SUM149 had high levels of c-Met. While the protein expression for survivin was characteristically high in some HER2+ BT cell lines (lanes 1-3), several TN cell lines, including HCC1187, MDA-MB468 and BT20 showed significantly high levels of survivin. We also observed a higher level of protein expression for TCF4, DVL, and Axin in most of the TN breast cancer cell lines tested as compared to the non-TN breast cancer cell lines.

### Differential expression of *beta-catenin* mRNA in patients with TN tumors as compared to the luminal and HER2-overexpressing tumors

**Figure 2:**
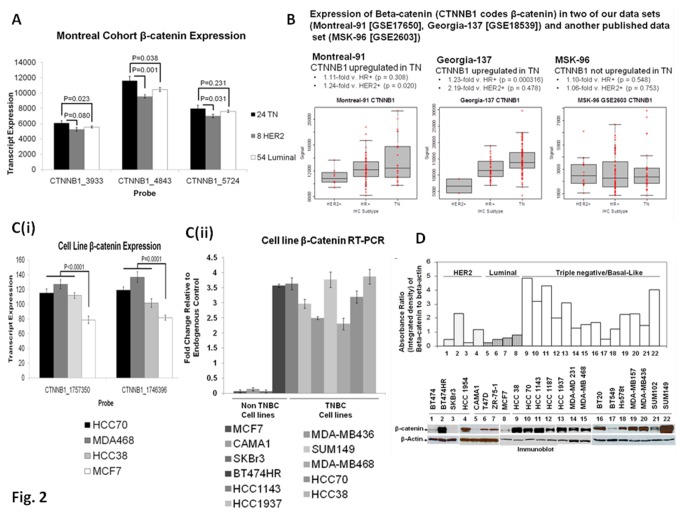
Expression of beta-catenin mRNA and protein in patients with TN breast tumors and cell lines. Expression of beta-catenin mRNA in patients with TN breast tumors (A), the expression of beta-catenin in two of our data sets (Montreal-91 [GSE17650], Georgia-137 [GSE18539]) and another published data set (MSK-96 [GSE2603]) (B), the expression of beta-catenin mRNA in different breast cancer cell line (C), and the expression of beta-catenin protein in different breast cancer cell line (D) are shown. (**A**) Expression of CTNNB1 probes that code the beta-catenin protein in the Montreal cohort. Mean expressions of three CTNNB1 probes (Montreal cohort) for TN (black), HER2+ (grey), and luminal (white) patients indicate greater levels of expression in the TN subtype as compared to luminal and HER2+ subtypes. Error bars represent one standard error of the mean and p-values are determined using t-test for unequal sample sizes and means. (**B**) Expression of beta-catenin (CTNNB1 encodes beta-catenin) with respect to immunohistochemistry (IHC) subtype in two of our data sets (Montreal-91 [GSE17650], Georgia-137 [GSE18539]) and another published data set (MSK-96 [GSE2603]). beta-catenin is up-regulated in TN as compared to the HER2+ group in the Montreal cohort (p = 0.020) and as compared to luminal (HR+) group in the Georgia-137 cohort (p = 0.000316) (C i). Expression of CTNNB1 in four BT cell lines. Expression of CTNNB1 in four cell lines, including three TN breast cancer cell lines HCC70 (black), MDA-MB-468 (dark grey), HCC38 (light grey), and one luminal-like cell line MCF7 (white). Values represent the mean of three replicates, and error bars represent one standard error of the mean. P-values are determined using a t-test for unequal sample sizes and means (C ii). RT-PCR expression of mRNA for beta-catenin in different BT cell lines (TNBC and Non-TNBC). Values represent the mean of three replicates, and error bars represent one standard error of the mean. (**D**) Differential expression of beta-catenin protein in different TN breast cancer cells (lanes 9-22) as compared to HER2+ (lanes 1-4), and luminal-like (lanes 5-8) breast cancer (BC) cell lines (beta-actin as the loading control). Upper panel shows the quantification of beta-catenin protein using imageJ program (absorbance ratio of beta-catenin to beta-actin).

Beta-catenin has been identified as a novel marker for BC [35]. Lin *et al*. showed that high beta-catenin activity significantly correlates with poor prognosis, and is a strong as well as an independent prognostic factor in BC [35]. Since beta-catenin is the functional readout integral to the WP signaling pathway, we set out to determine the status of expression of its mRNA in patient samples (Fig. 2A). In our study, beta-catenin (mRNA) is differentially expressed in TN tumors as compared to non-TN tumors. Our data (DASL-array) showed only a modest increase in the expression of beta-catenin (using three different probes; CTNNB1_3933, CTNNB1_4843, and CTNNB1_5724) in TN tumors as compared to the luminal and HER2-overexpressing tumors. We compared the levels of expression of beta-catenin mRNA in the tumor samples from St. Mary’s and Emory Cohorts as well as with a published data set (MSK-96 [GSE2603]) (Fig. 2B). Results show that the beta-catenin mRNA levels were significantly upregulated in tumor samples from TN patients as compared to tumor samples from HER2+ patients from St. Mary’s Cohort (*p* = 0.020). The beta-catenin mRNA levels were also significantly upregulated in the tumor samples from TN patients as compared to the tumor samples from HR+ patients from Emory Cohort (*p* = 0.000316) but not in the MSK-96 cohort of TN patients.

### Differential expression of *beta-catenin* mRNA and protein in cell line-based model of TNBC

Beta-catenin is the functional readout of WP. We studied the expression of beta-catenin (mRNA and protein levels) in TN breast cancer cell lines and compared it to the non-TN cell lines. The expression of mRNA for beta-catenin (CTNNB1_1757350 and CTNNB1_1746396) is higher in HCC70, MDA-MB468, and HCC38 TN breast cancer cell lines as compared to MCF7 (Fig. 2C i). Figure 2C ii showed mRNA expression of beta-catenin in TNBC and non-TNBC cells by RT-PCR. We also determined the protein levels of beta-catenin in 14 TN cell lines and compared them with 8 non-TN cell lines (4 HR+ cell lines and 4 HER2-amplified cell lines) (Fig. 2D). Densitometric analysis of the immunoblots clearly indicated that most of the tested TN cell lines have higher levels of beta-catenin protein (lanes 9-22) as compared to HER2+ BC cell lines and HR+ BC cell lines (lanes 1-4, and 5-8 respectively).

### Differential expression of *MMP7* mRNA in tumors from TNBC patients

**Figure 3:**
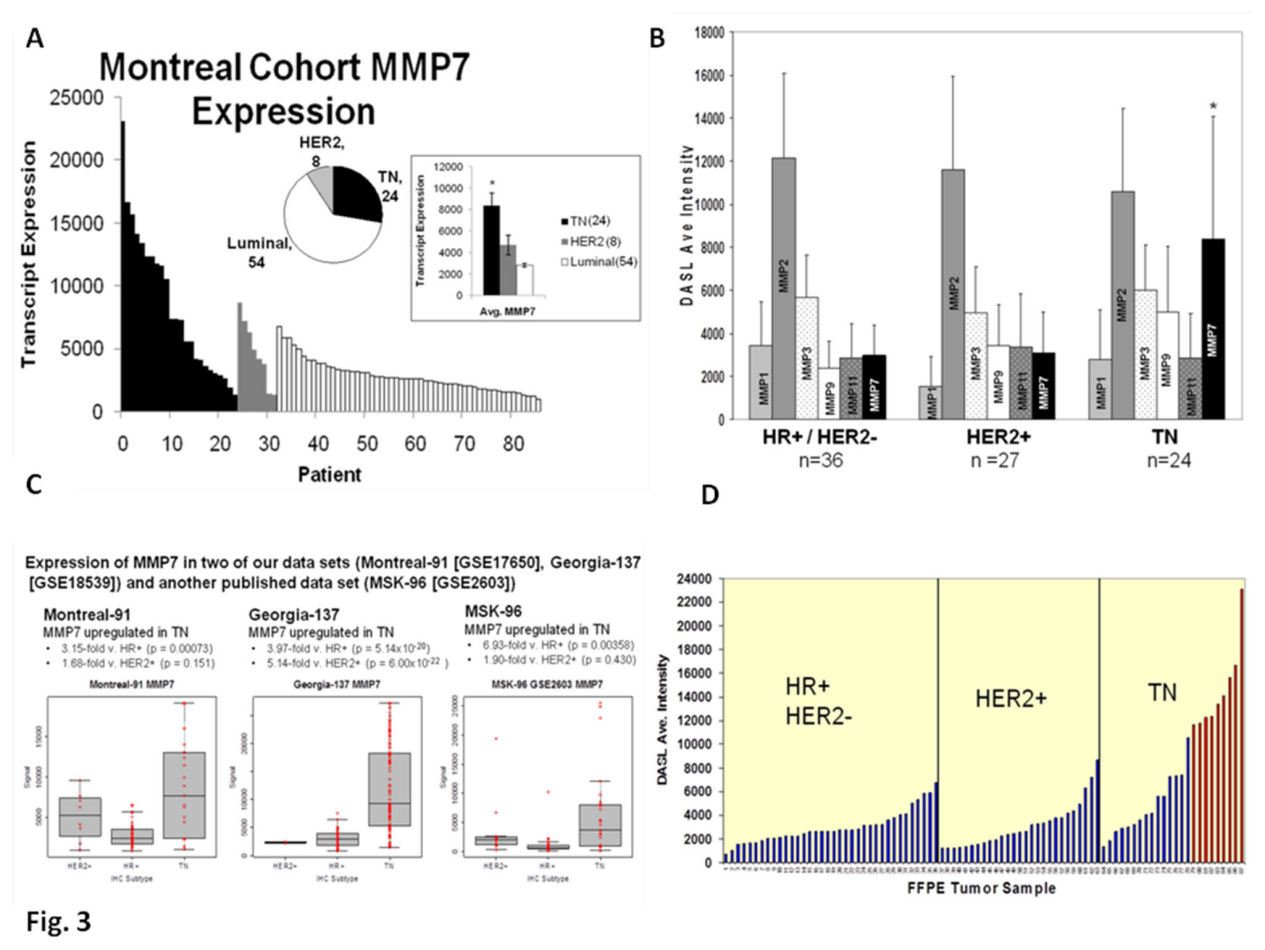
Expression of MMP7, one of the transcriptional targets of the WP as compared to other members of the MMP family in different subtypes of breast tumor samples from the Montreal and Georgia cohorts. (**A**) Figure shows MMP7 expression in the Montreal cohort. Expression of MMP7 is higher in the TN subtype. Expression is sorted from the highest to the lowest level for TN (black), HER2+ (grey), and luminal (white) breast tumor subtypes as classified by IHC. Inset shows average expression of MMP7 by subtype in the cohort. Average expression of MMP7 is significantly higher (P< 0.05) in the TN (black) subtype as compared to both the HER+ (grey) and luminal (white) subtypes as indicated by the asterisk. Numbers within bracket are the patients in each subtype. (**B**) Figure shows selective upregulation of mRNA for MMP7 in TN tumors of the Montreal cohort as evident from the DASL assay average intensity data for different metalloproteinases. Expression of MMP1, MMP2, MMP3, MMP9, MMP11 and MMP7 grouped by hormone receptor and HER2 status; HR+/HER2-, HER2+, and TN. HR+ (hormone receptor+) is ER+ and/or PR+; HER2+ is HER2-positive and HR±; TN is triple negative. Error bars represent STDEV. Level of transcript expression is represented by DASL average intensity. *P< 0.05, and n represents number of samples in each subset. (**C**) Figure shows the expression of MMP7 with respect to IHC subtypes in two of our data sets (Montreal-91 [GSE17650], Georgia-137 [GSE18539]) and another published data set (MSK-96 [GSE2603]). The expression of mRNA for MMP7 is significantly upregulated in TN as compared to the luminal subtype (HR+) in all the sets and HER2+ subtype in the Georgia-137 cohort (p = 6.00×10^-22^). The expression of the mRNA for the gene is significant in univariate analysis (p < 0.01). (**D**) The upregulation of MMP7 mRNA is observed in a subset of TNBC specimens as shown by the bar graph of the expression of MMP7 in each tumor in the cohort. Bars represent MMP7 mRNA expression in 87 FFPE breast tumor specimens (36 HR+/HER2- tumors, 27 HER2+/ HR± tumors and 24-TN tumors from the Montréal cohort) grouped by hormone receptor and HER2 status. MMP7 expression was upregulated in a subset ~40% (9/24) of TN tumor samples (shown in red) but not in any of the other (0/63) tumor samples (shown in blue).

Beta-catenin-mediated canonical activation of the WP occurs via beta-catenin mediated transcriptional upregulation of target genes, including MMP7 (Figure S2). Higher levels of expression of *MMP7* mRNA were observed in tumor samples from the TN patients as compared to HR+ and four HER2-amplified patients from St. Mary’s cohort (Fig. 3A). Figure 3B shows a selective upregulation of only MMP7 as compared to other members of the MMP family (*MMP1*, *MMP2*, *MMP3*, *MMP9*, *and MMP11*) in TNBC patients. When compared with our Emory cohort, and another published data set (MSK-96 [GSE2603]) (Fig. 3C), a similar trend of upregulation was observed in the TNBC subgroup as compared to the HR+ subgroups across all three sets. Interestingly however, when compared with the HER2+ subgroup only the Georgia set showed a highly significant increase (*p* = 6.00 ×10^-22^) in *MMP7*. Figure 3A shows the upregulation of *MMP7* in a subset of TNBC tumors representing approximately 40% (9/24) of all the TN tumor samples.

### Expression of MMP7 (mRNA and protein) and enzymatic activity of the secreted MMP7 in TN breast cancer cell lines

**Figure 4:**
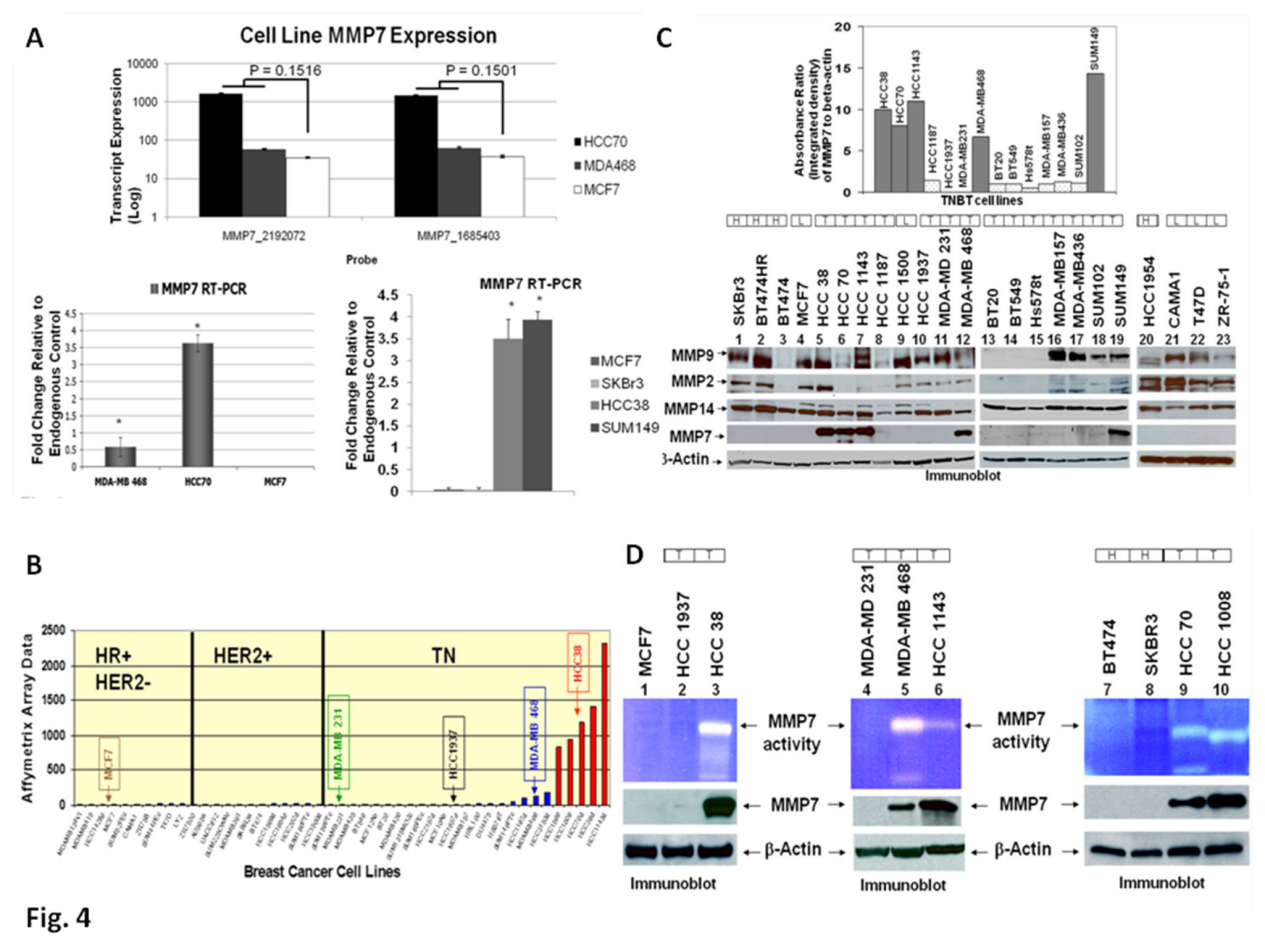
Expression of mRNA, protein and enzymatic activity of MMP7 in TNBC cell lines as compared to HER2+ and luminal BC cell lines. (**A**) Bar diagram (upper panel) shows mRNA expressions for MMP7 in HCC70 (black), MDA-MB468 (grey), and MCF7 (white) BT-like cell lines. Error bars represent one standard error of the mean and p-values are determined using a t-test for unequal sample sizes and means. Bar diagram in the lower panel (left) shows the validation of the expression of mRNA for MMP7 by RT-PCR in the BT cell lines. Expression of MMP7 measured by RT-PCR is concordant with microarray data for the HCC70, MDA-MB-468, and MCF7 cell lines. Expression is determined by the average of three replicates and bars represent one standard error of the mean (*p < 0.05). Bar diagram in the lower panel (right) shows RT-PCR expression of mRNA for MMP7 in different BT cell lines (MCF7, SKBr3, HCC38, SUM149). Values represent the mean of three replicates, and error bars represent one standard error of the mean (*p < 0.05). (**B**) MMP7 mRNA expression was upregulated in only a subset of TN breast cancer cell lines (shown in red) and not in any other types of BC-like cell lines. MMP7 affymatrix expression data from Neve et al. [36], grouped by hormone receptor and HER2 status. (**C**) Immunoblots showing differential expression of MMP7 protein (lanes 5-7, 12, 13, 14–16, 18-19) in TN breast cancer (T) cell lines as compared to HER2+ (H) and luminal (L) BC-like cell lines. Levels of MMPs 2, 9 and 14 are determined as references. Inset shows a differential expression of expression of MMP7 protein in a subset of TN breast cancer cell lines as determined by densitometry semi-quantitative analyses (imageJ program; absorbance ratio of MMP7 to beta-actin) of respective immunoblots. (**D**) Caesin-zymogram of the secreted-MMP7 and corresponding immunoblots of the cellular-MMP7 protein levels show both an increased expression and enzymatic activity of MMP7 (lanes 3, 5, 6, 9, 10) in TN breast cancer cell line (T) as compared to HER2+ (H) and luminal (L)-like cell lines.

We demonstrated significantly higher transcript expression for *MMP7* mRNA in TN cell lines as compared to non-TN cell lines (upper panel of Fig. 4A). Results were validated by RT-PCR (lower panel of Fig. 4A). Fig. 4B shows that *MMP7* mRNA expression is upregulated in only a subset of TN cell lines and not in the other BT cell lines [from the data set of [36]. *MMP7* mRNA expression was very high in HCC70, HCC38, HCC1143, and HCC1599 TN cell lines (as also observed in the case of expression of protein levels; Fig. 4C) in contrast to the low expression levels of *MMP7* in MDA-MB468, HCC1187, and SUM149 TN cells, and almost undetectable levels of *MMP7* in several other TN cell lines, including MDA-MD231, BT549, BT20, HCC1937, and Hs578t. We also compared the expression of the MMP7 protein in various TN cell lines with the expression of some of the members of the MMP family, including MMP2, MMP9 and MMP14 (Fig. 4C). Although, other members of the MMP family were expressed among different BT cell lines, MMP7 was found to be differentially expressed exclusively in TN cell lines. Interestingly, we found that there was a cell-line specific expression of MMP7 within the TN subtype. HCC38, HCC70, HCC1143, MDA-MB468, and SUM149 express higher levels of MMP7 protein (as well as a functionally active form of the secreted enzyme) as compared to other TN cell lines (inset of Fig. 4C). Since TN tumors can be aggressive and secreted-MMPs are critical in the micro-dissemination of tumor cells, we also tested the enzymatic property of the secreted MMP7 by casein zymography (for assessing the specific enzyme activity of the secreted MMP7) in TN cell lines. HCC38, HCC70, HCC1143, and MDA-MB468 TN cell lines secreted active MMP7. Non-MMP7 expressing TN cell lines (MDA-MB231 and HCC1937) and non-TN cells (MCF7, BT474, and SKBr3) were used as negative controls for the immunoblot and zymograph (Fig. 4D). Taken together, our cell line data recapitulates our data from tumor samples in a manner that MMP7 expression (mRNA/protein/enzyme activity of the secreted protein) is differentially upregulated in only TN breast cancer cell lines in a subtype specific manner, and more interestingly in a sub-population of TN cell lines.

### Higher levels of *MMP7* mRNA in a sub-population of TNBC patients is associated with lower levels of *PTEN* mRNA

**Figure 5:**
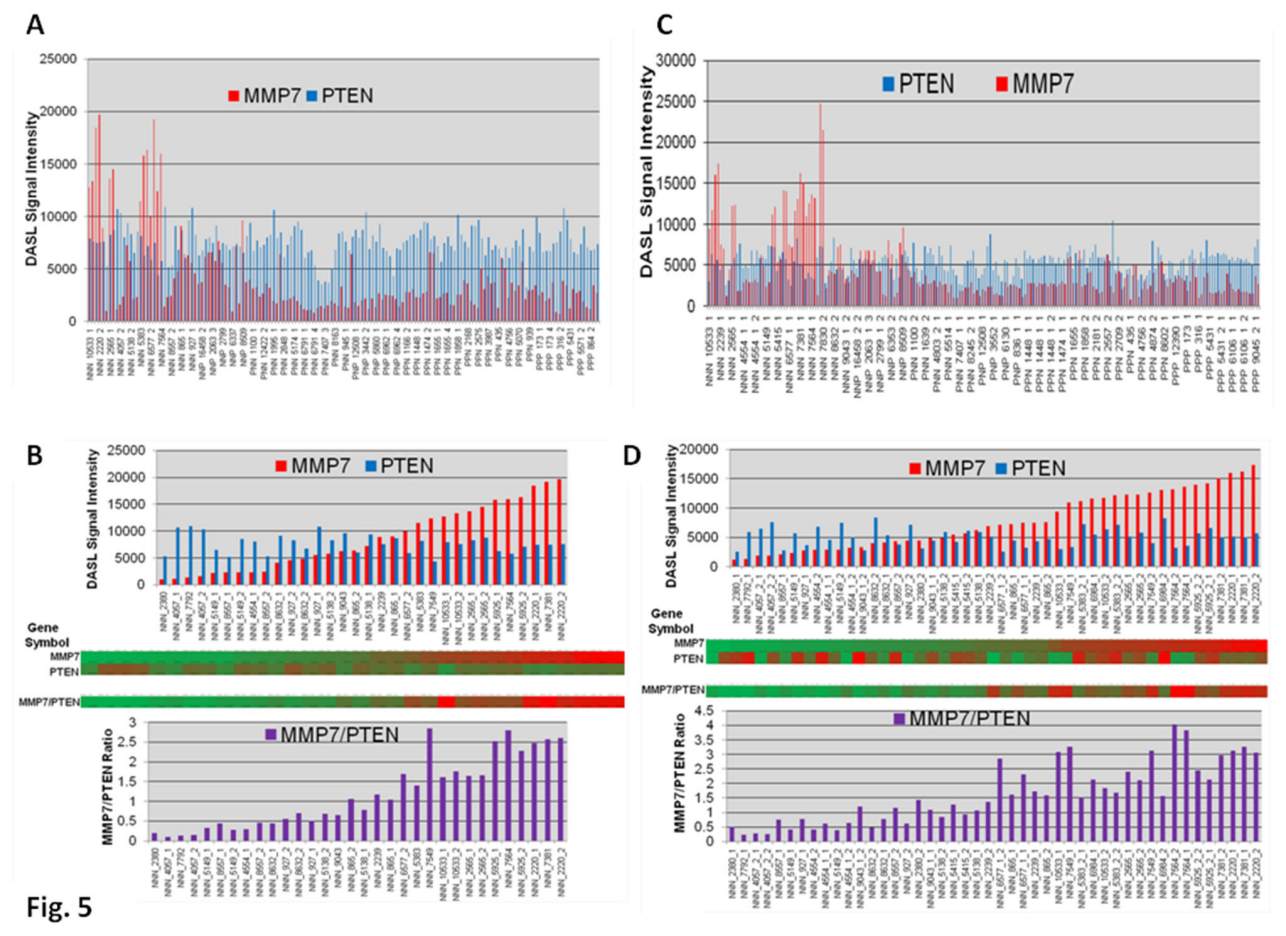
Relative levels of expression of MMP7 and PTEN mRNAs from tumors of Montreal cohort. Relative levels of expression of MMP7 and PTEN mRNAs (DASL intensities) using standard (A, B) as well as custom (C, D) panels from tumors of the Montreal cohort Data show (**A** & **C**) a differential expression of high MMP7 in the TN (NNN) tumors as compared to HR+(PPP, PPN, PNN) and HER2-enriched (NNP, PNP) groups. Analyses of levels of expression of MMP7 to the PTEN mRNAs (DASL intensities) in TN samples show that (1) MMP7 mRNA is expressed in only a subset of TN tumors (**A** & **C**), and (2) the presence of a higher level of MMP7 within the individual tumors of TN subset are associated with relatively lower levels of PTEN mRNA (**B** & **D**). These reciprocal levels of expression of MMP7 to the PTEN mRNAs (DASL intensities) were observed from the MMP7 to PTEN ratios, and heat maps of expressions in both standard (**B**) as well as custom (**D**) panels of the Montreal cohort.

Figure 5 shows the levels of expression of *MMP7* and *PTEN* mRNAs (DASL signal intensities) using standard (Figure 5 A, B) as well as custom (Figure 5C, D) panels from tumors of the Montreal cohort. Differential expressions of high *MMP7* levels were observed in the TN (NNN in the figure) tumors as compared to HR+ (PPP, PPN, PNN in the figure) and HER2-enriched (NNP, PNP in the figure) groups. We analyzed the levels of mRNA expression of *MMP7* and *PTEN* (DASL intensities) within TN group. Having confirmed the earlier finding that *MMP7* mRNA is expressed only in a subset of TN tumors, we showed that high-*MMP7* expressing TN tumors had relatively low-*PTEN* mRNA levels. These reciprocal levels of expression of *MMP7* to the *PTEN* mRNAs (DASL intensities) were also evident from the plots of *MMP7* to *PTEN* ratios (lower panels of Figure 5B, D), and heat maps (middle panels of Figure 5B, D) of expressions in both standard (Figure 5A, B) as well as custom (Figure 5C, D) panels of the Montreal cohort.

### TN breast cancer cells with high levels of secreted MMP7 are null for functional PTEN

**Figure 6:**
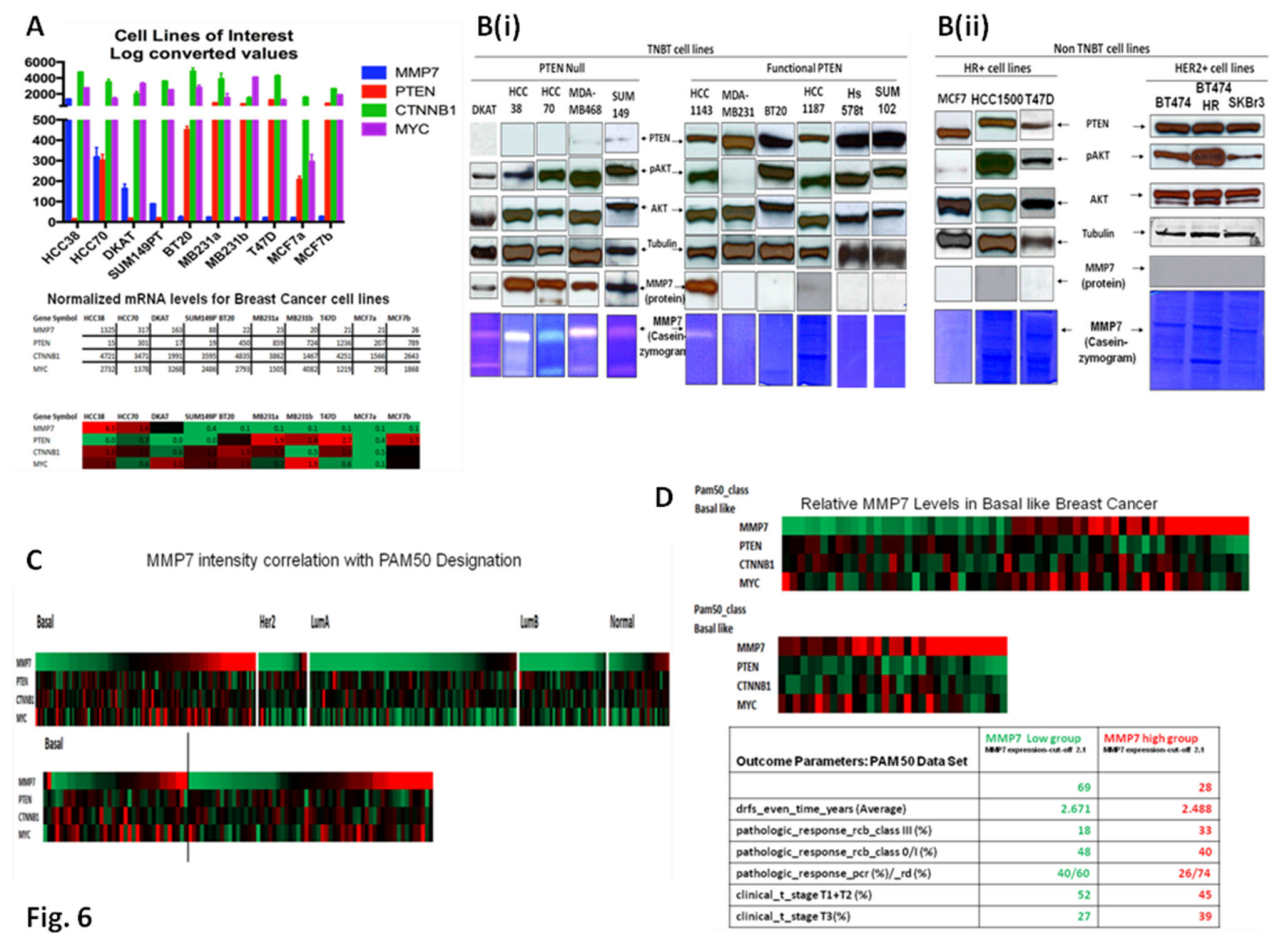
Relationship between MMP7 expression and PTEN expression in TN tumors and cell lines, and MMP7 intensity correlation with PAM50 designation. Relationship between MMP7 expression and PTEN expression in TN tumors and cell lines (A and B), and MMP7 intensity correlation with PAM50 designation (C and D) are presented. **A**. Cell lines were downloaded from GEO (GD3285) with replicates averaged for each cell line. HCC38 and SUM149PT did not have replicates. Error bars represent the coefficient of variation between each of the replicates. MDA-MB231 and MCF7 cell lines had two distinct groups of replicates that are included on this graph to demonstrate the variability standard cell lines can have (most likely due to passage number differences). Relative levels of expression of MMP7 and PTEN mRNAs (published DASL intensities) from different TN cell lines showing a differential expression of high MMP7 in the TN (HCC38, HCC70, MDA-MB231, BT20, DKAT and SUM149T) cell lines as compared to non-TN cell lines (MCF7, T47D) as shown in the upper panel. Normalized mRNA levels for breast cancer cell lines show that the presence of a higher level of MMP7 mRNA in the individual TN cell lines is associated with relatively lower levels of PTEN mRNA. These reciprocal levels of expression of MMP7 to the PTEN mRNAs (DASL intensities) were observed in heat map of gene expression as shown in the lower panel. Cell line intensity data generated from Affymetrix Gene Expression arrays (Upper panel). RMA normalized intensity values shows relative inter-gene levels of each mRNA in a specific cell line with the (Lower panel) heat map using Z’ values calculated for each specific gene to visualize intra-gene mRNA levels. **B (i)**. Expression of MMP7 protein and the secretion of functional MMP7 were determined in the PTEN-null and PTEN expressing TNBC cells. High levels of expression of MMP7 protein and the secretion of functional MMP7 (casein zymogram from the conditioned media) in TNBC cells is inversely related to the presence of functional PTEN (protein expression as well as pAKT). **B (ii)**. Expression of MMP7 protein and the secretion of functional MMP7 were determined in the PTEN expressing non-TNBC cells (HR+ and HER2 over-expressed). **C**. Patient downloaded from GEO (GSE25055). Top panel showing PAM50 designations used to separate the patients into each subtype. Each subtype was then sorted from left to right by increasing MMP7 intensity. Bottom panel is sorted by increasing MMP7 from left to right. Bold vertical line separates pCR on the left with residual disease (RD) on the right. **D**. Heat Map of expression of mRNAs for MMP7, PTEN, CTNNB1, c-MYC genes (PAM50 data) in Basal, HER2E and HR+ subsets of BC. Table (Lower panel) shows the outcome data for triple negative breast cancer patients from PAM50 data set with high MMP7, and low MMP7. Patient data from Fig. 6C were further examined for basal-like subtype using Z’ scores. Top panel shows increasing MMP7 Z’ score from left to right for all patient samples. Middle panel displays only patients with Z’ scores over 2.0 for MMP7, which shows the association with high MMP7 levels and low PTEN levels. The outcome data for the patients expressing low-MMP7 and high-MMP7 in their tumors are shown in the table (Lower panel).

Figure 6A shows heat map of expression of mRNAs for *MMP7*, *PTEN*, (Affy data) in BT cells. Normalized mRNA levels for BC cell lines show that the high-*MMP7* expressing TN breast cancer cells (HCC38, HCC70, DKAT, and SUM149) have significantly lower levels of *MMP7* mRNA as compared to the other low-*MMP7* expressing TN cells as well as no-*MMP7* expressing non-TN cells (MCF, T47D). To further investigate the association between MMP7 and PTEN gene expressions in TNBC, we tested the functions of the secreted MMP7, and determined downstream signals PTEN in high-MMP7 expressing TNBC cells. Figure 6B shows that high-casein zymograph positive (functional read out of the secreted MMP7) DKAT, HCC38, HCC70, MDA-MB468 and SUM149 TNBC cells do not express PTEN protein, and have high levels of pAKT (immediate downstream effecter of PTEN). In contrast, functional PTEN expressing TNBC cells (MDA-MB231, BT20, HCC1187, Hs578t, and SUM102) do not express MMP7 (protein or enzyme activity; fig. 6Bi). Although, PTEN-positive HCC1143 cell lines expressed MMP7 protein, it showed a limited enzymatic activity in casein zymograph. Interestingly, non-TNBT cell lines (both HR+ cells like MCF7, HCC1500, T47D, and HER2+ cells like BT474, transtuzumab-resistant BT474HR, SKBr3) expressing functional PTEN have shown no MMP7 (Fig 6B ii).

We have found a negative correlation between MMP7 and PTEN expression levels in cell line experiments. For all cell lines tested (Fig. 6B) the correlation coefficient is -0.47. When further examined by highly expressed MMP7 and low expressed PTEN the correlation coefficient is -1.0 (HCC38 and DKAT cell lines with replicates).

### Higher levels of *MMP7* mRNA in a sub-population of TNBC patients are associated with poor outcome parameters

We decided to further investigate using data from human tumor samples (GSE25055) to confirm our findings. When examining data from TNBC / Basal subtype with high *MMP7* expression and low *PTEN* expression we found a correlation coefficient of -0.54. This combined with the outcome data for these samples suggests that our assertions of *MMP7* and *PTEN* levels provide a molecular subtype with a predictive signature for pCR.

To understand the clinical relevance of high *MMP7* expression in TNBC patients, we studied the outcome data in high-*MMP7* expressing patients using PAM50 data set (Figure 6 C, D). First we validated our observations (differential upregulation of *MMP7* in a sub-population of TNBC tumors) in PAM50 data set, and then we sought to understand the relationship between the subpopulation of TNBC patients expressing high level of *MMP7* mRNA and the patient outcome. Patient data were downloaded from GEO (GSE25055). Top panel (Figure 6C) showed PAM50 designations used to separate the patients into each subtype. Each subtype was then sorted from left to right by increasing *MMP7* intensity. Bottom panel (Figure 6C) was sorted by increasing *MMP7* from left to right. Data from patients’ samples (Figure 6C) were further examined for basal-like subtype using Z’ scores. Top panel (Figure 6D) showed an increasing *MMP7* Z’ score from left to right for all patient samples. The association between outcome parameter, and *MMP7* levels is presented in the table (lower panel, Figure 6D). High-*MMP7* (*MMP7* expression-cut-off 2.0) group of patients exhibited comparatively lower PCR (26%) than low-*MMP7* group (40%). In contrast, high-*MMP7* expressing patients exhibited comparatively higher RD (74%) than low-*MMP7* group (60%). Interestingly, pathologic_response_rcb_class III (%) was significantly higher (33) in high-*MMP7* group as compared to low-*MMP7* group (18). Although patients in clinical_t_stage T1+T2 (%) did not differ much between high-*MMP7* group and low-*MMP7* groups, the high-*MMP7* group had more patients (39) in clinical_t_stage T3 (%) than the low-*MMP7* group (27).

### An attenuation of WP caused downregulation of MMP7 and enzymatic function of the secreted MMP7 in TNBC cells

**Figure 7:**
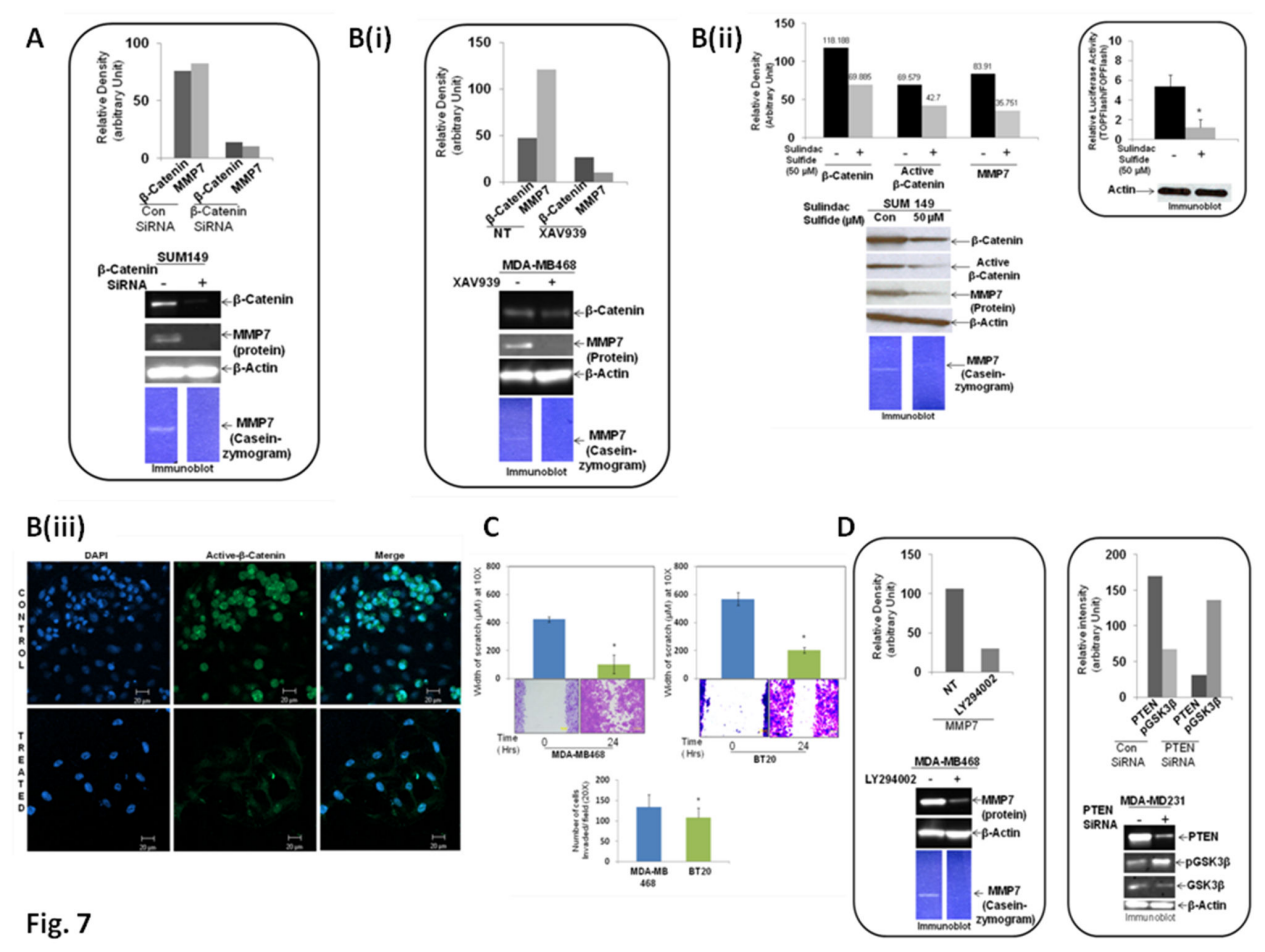
An attenuation of WP caused downregulation of MMP7 in PTEN null TNBC cells. PTEN null MMP7 expressing TNBC cell line, SUM149 was transiently transfected with beta-catenin SiRNA and MMP7 protein and its enzymatic activity were determined by Western blot and casein zymography respectively (**A**). Upper bar diagram showed the relative desitometric expressions of beta-catenin and MMP7. Pharmacological attenuation of beta-catenin in PTEN null MMP7 expressing TNBC cell lines, MDA-MB468 (B i) and SUM149 (B ii) following the treatment with XAV939 (B i) and sulindac sulfide (B ii) caused a decrease in MMP7 protein expression as well as an abrogation of its enzymatic activity. Upper bar diagrams showed the relative desitometric expressions of beta-catenin, active beta-catenin and MMP7. Relative luciferase activity (TOP Flash over FOP Flash) measured in SUM149 cells following sulindac sulfide was plotted (three different experiments). Error bars represent standard error of the means (SDs), and statistical significance was determined by paired t-test. *P < 0.05. The lower panels showed images of Western blot measuring β-actin (Inset of B ii). Sulindac sulfide treatment substantially reduced nuclear immune-fluorescence of beta-catenin (active form) in TNBC cells (B iii). To find out the involvement of MMP7 in metastasis-associated phenotypes, fibronectin-directed migration (C, upper panel) and matrigel-invasion (C, lower panel) were determined in MMP7 expressing MDA-MB468 cells and MMP7 null BT20 cells. Error bars represent standard error of the means (SDs), and statistical significance was determined by paired t-test. *P < 0.05. Since the pan PI3K inhibitor, LY294002 has been shown to closely mimic the effects of PTEN reconstitution in PTEN-deficient cancer cells, to understand the role of PTEN on the expression/enzymatic activity of MMP7 in TNBC cells, we have treated PTEN null MMP7 expressing MDA-MB468 cells with LY294002 (**D**). Data showed a decrease in MMP7 following LY294002 treatment in MDA-MB468 cells.

Having observed that higher levels of *MMP7* mRNA in a subpopulation of TNBC patients are associated with poor outcome parameters, we conducted a number of cell lines-based experiments to seek a causal relationship between beta-catenin and MMP7 levels in TNBC cells. Our data showed that a decrease in cellular beta-catenin caused down-regulations of MMP7 levels in PTEN null TNBC cells. Figure 7 showed that a decrease in beta-catenin level following, (1) genetic manipulation (beta-catenin SiRNA), (2) pharmacological attenuation (XAV939, WP-modulator that stabilizes axin) [28,29], or (3) a decrease in the functional readout of the WP (beta-catenin) by sulindac sulfide [30] in PTEN null TNBC cell lines (SUM149 and MDA-MB468) caused a decrease in both protein levels as well as enzymatic activity of MMP7. Inset of Fig. 7B (ii) showed the decrease in the transcriptional activity of beta-catenin following sulindac sulfide treatment in TNBC cells which corroborates with the decrease in the active form of nuclear-beta-catenin (immunofluorescence) (Fig. 7B iii). Taken together, our data demonstrate that WP transcriptionally regulates MMP7 expression and the enzymatic function of the secreted MMP7 in TNBC cells via active-beta-catenin. Recently, we have observed that Wnt signaling in TNBC is associated with high metastasis in patients with poor prognosis [19]. Therefore, we tried to understand the role of MMP7 in TNBC cells in the context of metastasis-associated phenotypes, integrin-directed migration and matrigel-invasion. Figure 7C showed that MMP7 expressing MDA-MB468 cells exhibit higher rates of migration as well as invasion than MMP7-null BT20 cells.

Since we observed a negative correlation between PTEN and MMP7 levels in both tumor samples from patients and in TNBC cell lines, we tried to understand the mechanistic relationship between PTEN and MMP7 by pharmacologically mimicking the PTEN’s presence by treating the cells with a pan PI3K inhibitor, LY294002 in MMP7 expressing MDA-MB468 TNBC cells. Studies by Su et al., demonstrated that LY294002 closely mimics the effects of PTEN reconstitution in PTEN-deficient cancer cells [37]. To understand the role of PTEN on the expression/enzymatic activity of MMP7 in TNBC cells, we have treated PTEN null MMP7 expressing MDA-MB468 cells with LY294002 (Figure 7D left panel). Figure 7D left panel showed that LY294002 treatment abrogated both the cellular levels of MMP7 and the enzymatic activity of the secreted MMP7. Furthermore, we also carried out experiments with PTEN SiRNA to know the mechanistic relationship between PTEN null-ness and high MMP7 expression in TNBC cells. SiRNA-mediated attenuation of cellular levels of PTEN protein in TNBC cells in our experiments resulted in phosphorylation-mediated inactivation of GSK-3β (Figure 7D right panel), which controls proteosome–mediated degradation of beta-catenin. Since inactivation of GSK-3β would inhibit degradation of beta-catenin (Figure S2), these data imply that a decrease in PTEN levels would transcriptionally activate MMP7 via GSK-3β-beta-catenin-TCF pathway.

## Discussion

The arsenal of “targeted therapy” for TNBC has been inadequate. Therapeutic options for the patients were limited due to the lack of identification of pathway-specific targets [4,38,39], and the absence of a validated targeted-therapy [40,41]. It is anticipated that a greater understanding regarding (1), the molecular complexity of the biology of TNBC, and (2) its heterogeneity based molecular stratification/ sub-classifications might reveal novel targets and identify markers around which the future clinical trials in a molecularly defined sub-group could be premeditated [42–45]. Our study pointed out an up-regulation of the WP as a key expression signature of TNBC, and revealed for the first time that the high expression of MMP7, a transcriptional target of WP, in a subpopulation of TNBC patients has been associated with the loss of tumor suppressor PTEN, the most common first somatic event associated with basal-like subtype.

An association of genetic variation in genes implicated in the beta-catenin destruction complex with risk of breast cancer has been reported [46]. Yang *et al* reported that FZD7-involved canonical Wnt signaling pathway is essential for tumorigenesis of TNBC, and FZD7 showed promise as a biomarker and potential therapeutic target for TNBC [47]. The heatmap presented in the supplemental data (Figure S4) agrees with our previous observation [19] (*MS resubmitted*), and a report by Khramtsov *et al.*, which states the association of Wnt signaling in TNBC with poor prognosis and metastasis [48]. Reis-Filho’s group identified that WP activation in BC has been associated with the TN phenotype but not with CTNNB1 mutation [15]. Our report agrees with their results wherein they revealed that a lack of or a reduction of beta-catenin membrane expression and/or nuclear accumulation were significantly associated with estrogen receptor negativity, absence of *HER2* gene amplification, lack/reduction of E-cadherin expression and tumors of the TN and basal-like phenotype, indicating that WP activation is preferentially found in TN/basal-like breast carcinomas, and is associated with poor clinical outcome.

We observed a modest increase in the mRNA levels of beta-catenin in TNBC samples as compared to the non-TNBC samples. *Beta-catenin* mRNA levels were marginally higher in two patient sample sets but not a third one (Fig. 2B). Similarly, *beta-catenin* mRNA levels were marginally higher in TN breast cancer cell lines compared with MCF7 (Fig. 2C). The nuclear and the cytosolic accumulation of beta-catenin is the read-out for WP (canonical) activation (Figure S2), and the activation of the WP inhibits beta-catenin degradation [48,49]. Thus, mRNA status may not always truly represent the activation state of the pathway. This argument is further supported by a report by Lopez-Knowles *et al*., who reported that when cases were distributed into the five phenotypes of BC, a high membranous beta-catenin H1 score was associated with the basal-like subtype (*p* = 0.008). A high cytoplasmic beta-catenin H2 score was also associated with the basal-like (*p* < 0.0001) and HER2 (*p* = 0.01) subtypes, and a low cytoplasmic expression (*p* < 0.0001) was associated with the luminal A subtype [50]. In contrast to *beta-catenin* mRNA levels, beta-catenin protein expressions were significantly greater in TN cell lines compared with other two subtypes.

The expression of MMP7 is primarily regulated at the transcriptional level (see 51. *MMP7* mRNA was detected in the neoplastic epithelial tumor cells of 70-91% of breast adenocarcinomas, and the expression of MMP7 in the mammary epithelium was known to contribute to the early-stage mammary tumorigenesis [52]. A study by Jiang *et al.*, reported that MMP7 is expressed aberrantly in human breast tumors [53]. We observed (Fig. 3C) that in Emory cohort the expression of mRNA for *MMP7* was significantly higher in tumors from patients with TNBC as compared to tumor samples from, (a) HR+ patients (3.97 fold; *p* = 5.14 ×10^-20^), and (b) HER2+ patients (5.14 fold; *p* = 6.00 ×10^-22^). Currently, we are in the process of understanding the reasons for this discrepancy in light of differences in ethnicity of the TNBC patients, and whether or not it can be explained by the enrichment of the Georgia sample set with African American patients [54,55].

The upregulation of *MMP7* mRNA is observed in a “subset” of TNBC specimens (Fig. 3D). To the best our knowledge, this is the first observation of an “MMP7-enriched sub-population” within the TN subtype of BC. Interestingly, our cell line data (protein expression and enzyme activity) also demonstrates that there is an MMP7-enriched sub-population of TN breast cancer cell lines recapitulating the patient breast tumor data (Fig. 4). Recently, Lehmann *et al.*, analyzed gene expression profiles from 21 BC data sets and conducted cluster analysis (587 TNBC cases) to identify 6 TNBC subtypes displaying unique gene expression [56].

We have used BT cell lines to model breast tumors [57,58]. Although BT cell lines are sometimes genetically distinct from the tumors, they are reported to retain the expression patterns with relevance to the luminal-basal subtype distinction [36]. A study by Lin *et al.*, reported a correlation between cyclin-D1 expression and beta-catenin/Tcf4 activity in BC cell lines [35] wherein, a transactivation of beta-catenin was correlated with cyclin-D1 expression both in cell lines and in patient-samples. We also observed a positive correlation between the beta-catenin and cyclin-D1 protein levels in TN cell lines, including HCC38. Benhaj *et al.*, have already reported the expression (PCR) of Wnt-ligands, FDZ-receptors, and LEF-TCF transcription factors in BT cell lines [59]. We report a higher expression of TCF4 in MDA-MB468 and BT20 cells as compared to MCF7 and BT474 cells. Similarly, we observed an increase in FZD7 in TN patient samples that is comparable with the expression of the protein in both MDA-MB468 and BT20 cell lines (in contrast to MCF7 and BT474 cells), which is in line with our result. In the case of Wnt-ligands, similar trends of expressions for Wnt3A and Wnt7B in MDA-MB468 cells were observed as compared to MCF7 cells (Figure S5). Interestingly, it has been reported that Wnt3 is a ligand of FZD7 [60].

In our results, most of the TN cell lines expressed higher levels of TCF4, DVL, and axin as compared to non-TN cell lines. A few exceptions to this observation were the BT474HR, HCC1954 and T47D cell lines, which showed comparatively higher levels of TCF4 and beta-catenin (Figure S6 & Fig. 2D). High levels of beta-catenin protein in trastuzumab-resistant (BT474HR), *PIK3CA* mutation carrying HER2-overexpression (HCC1954), and *PIK3CA* mutation carrying HR+ (T47D) cell lines might be the result of the constitutively high levels of p-AKT in these cells (Figure S1) that in turn could have influenced the degradation of cytosolic beta-catenin via p-GSK3β (via beta-catenin destruction complex) [49]. Recently, expression of Wnt3 has been shown to activate WP and promoted EMT-like phenotype in trastuzumab-resistant HER2-overexpressing breast cancer cells [61]. The higher levels of TCF4 in BT474HR cells can be also explained by their higher expression of beta-catenin. The TCF4 promoter contains a single consensus TCF-binding site critical for activation by beta-catenin, which can directly induce transcription from the TCF4 promoter [62]. Similarly, we observed that TN breast cancer cell lines (HCC38, HCC1143 and SUM149) with very high expression of beta-catenin also showed appreciable levels of TCF4 protein expression (Figure S6 & Fig. 2D). Sievers et al. studied absolute beta-catenin concentrations in WP-stimulated and non-stimulated cells (including BT cell lines) and found that the intracellular beta-catenin concentration depends on the mode and level of activation of the WP [63]. We found a wide variation in the levels of expression of the beta-catenin protein among different TN cell lines, which presumably reflects the variable state of WP activation in these cells. We showed a higher level of active beta-catenin immunofluorescence in the nucleus of MDA-MB231 TN cells as compared to non-TN cell lines [31]. The observed localization of higher levels of the active (dephosphorylated) beta-catenin in the nucleus of MDA-MB231 TN breast cancer cells may indicate that the nuclear/cytosolic ratio of beta-catenin can be more accurately associated with the state of activation of the WP (data not shown), and to the outcome in TNBC patients [19]. Khramtsov *et al.*, have also reported an association between nuclear and cytosolic localization of beta-catenin protein with other markers of the basal-like phenotype, including hormone receptor/HER2 negativity, cytokeratin 5/6, vimentin expression, and stem cell enrichment [48]. Our data show a cell-line specific expression of MMP7 (mRNA and protein) within the TN subtype. HCC38, HCC70, MDA-MB468, and SUM149 cells expressed higher levels of functionally active secreted MMP7 protein as compared to other TN cell lines (Fig. 4). A similar differential expression of *MMP7* was observed in the patient population (Fig. 3).

We observed a low or no expression of *PTEN* mRNA in the high-*MMP7* expressing TNBC tumors as well as TNBC cell lines. Previous studies indicated the ability of PTEN to regulate beta-catenin in cancer cells. A constitutively high concentrations of nuclear beta-catenin was reported in PTEN-null prostate cancer cell lines, and a re-expression of PTEN in those cells resulted in activation of GSK-3β and degradation of beta-catenin [64]. We also demonstrated (Fig. 7D right panel) that SiRNA-mediated attenuation of cellular levels of PTEN protein in TNBC cells led to an increased level of phosphorylation of GSK-3β (which regulates proteosome–mediated degradation of beta-catenin) indicating a mechanistic relationship between PTEN’s function and the expression of MMP7 via GSK-3β-beta-catenin-TCF pathway. The clinical significance of a negative correlation between PTEN expression and MMP7 expression is reported in NSCLCs [65], gastric cancer [66],, And colorectal cancer [67]. MMP7 is a target of beta-catenin transactivation in certain tumors [68] and evidence show the involvement of MMP7 activation in human cancer metastases [69]. Expression of MMP7 was one of the significant predictors of lower patient survival, and MMP7 were significantly related to liver metastasis in colon cancer indicating that MMP7 overexpression appears to be a biological marker of aggressive phenotype in colon carcinoma [70]. Since the PAM50 gene-set is often used for gene expression-based subtyping [71], we used this gene-set to investigate the clinical significance of our observation using the GSE25055 cohort. We observed that the pathologic_response_rcb_class III (%) was higher (33%) in the high-MMP7 group than in the low-MMP7 group (18%). Zheng et al. observed that MMP7 expression was positively associated with tumor size, Borrmann’s classification, invasive depth, metastasis and TNM staging in gastric carcinoma [66]. A similar role of MMP7 in vessel invasion and metastasis is reported by studying the expression and tissue localization in human gastric carcinoma [72]. We observed that among patients with the clinical_t_stage T3 (%), 39% exhibited high-MMP7 (39%) while 27% exhibited low-MMP7 (Fig. 6B). A study by Jiang et al. reported that the highest level of MMP7 was observed in high-grade breast tumors and that from patients with moderate and poor prognosis [53]. Although they have not studied the subtype specific distribution of MMP7, their study showed that high levels of MMP7 were significantly linked with poor long-term survival (*p* = 0.0143).

Zheng et al. showed a positive correlation of MVD (microvessel density) with tumor size, invasive depth, metastasis and TNM staging, and MVD depended on decreased PTEN expression and increased MMP7 expression. Interestingly, we observed a higher % of RD in the high-MMP7 group. Presence of this negative correlation between *PTEN* expression and *MMP7* expression together with the observed low pathologic_response_pcr (%) as well as the High Rd (%) in the high-*MMP7* expressing patients in our result might be an indication of a clinically identifiable subclass within this subtype in which MMP7 and PTEN might play a role in the context of tumorigenesis, and disease progression. Since loss of PTEN is the most common first event and is associated with basal-like subtype [9], and MMP7, a secreted matrix-metalloproteinase required for cell invasion, is one of the transcriptional targets of WP, our observation of down-regulated PTEN expression in high MMP7 expressing tumors from TNBC patient might provide a novel insight into the regulatory effects of PTEN on MMP7 expression in TNBC, and could serve as an important biological indicator of the behavior of these TNBC cells.

Attenuation of WP by genetic and pharmacological manipulation of beta-catenin levels in MMP7 expressing PTEN null TNBC cells in our study caused a downregulation of MMP7 protein levels and enzymatic activity. MMP7 is one of the target genes of the APC–beta-catenin–TCF Pathway [68]. The transcription regulation of matrilysin requires the LEF-1/TCF beta-catenin pathways [68] and a correlation between beta-catenin widespread nuclear expression and MMP7 overexpression was reported in sporadic desmoid tumors [73]. Beta-catenin is knowm to regulate the expression of the MMP7 in human colorectal cancer [74]. Members of MMPs family, including MMP7 play a distinct role in tumor cell metastasis via metrix degradation [75] and integrin-mediated migration along with invasion are critical steps for the metastasis of tumor cells. We observed that MMP7 expressing MDA-MB468 cells exhibits higher migratory and invasive property than MMP7 null BT20 TNBC cells. In gastric carcimona, MMP7 expression was positively associated with tumor size, while negatively associated with PTEN expression [66]. Recently, Bi et al., reported a significant negative correlation between MMP7 and PTEN expression in colorectal cancer (r=-0.403, p>0.05) wherein they observed that a reduced PTEN expression and MMP7 high expression play important roles in the pathogenesis of colorectal cancer [67]. Examining data from TNBC / Basal subtype (from human tumor samples; GSE25055) with high MMP7 expression and low PTEN expression we found a correlation coefficient of -0.54. This combined with the outcome data for these samples suggests that our assertions of MMP7 and PTEN levels provide a molecular subtype with a predictive signature for pCR. To understand the functional relationship between PTEN null-ness and high expression of MMP7, we tested the consequence of PTEN mimicking effect of LY294002 in PTEN null TNBC cells. Su et al., demonstrated that LY294002 closely mimics the effects of PTEN reconstitution in PTEN-deficient cancer cells [37]. Our result that LY294002 treatment abrogated MMP7 expression in MDA-MB468 cells further substantiates the inverse functional relationship between PTEN and MMP7.

In light of reports from Reis-Filho’s team [15] who established a preferential increase in beta-catenin (IHC) in TNBC patients, and Kornelia Polyak’s team [9] who identified PTEN loss as the most common first somatic event associated with basal-like subtype, here we report for the first time that the functional upregulation of MMP7, a transcriptional target of beta-catenin is associated to the loss of PTEN gene function. Our data categorize the activation of the WP by molecular subtypes of BC, and associates MMP7, one of the target genes of the WP to PTEN loss, the most common first somatic event associated with basal-like subtype suggesting that the WP pathway can provide attractive pharmacological targets for TNBC. We are currently studying the functional relevance of this pathway in the regulation of metastasis-associated tumor cell phenotypes in TNBC.

## Supporting Information

Figure S1 **Expression of the levels of pAKT in trastuzumab sensitive (**S**) and resistant (**R**) HER2+ breast cancer-like cell lines.** Levels of pAKT were determined by immunoblot from two (SKBr3 and BT474) trastuzumab sensitive (S) and two (BT474HR and HCC1954) resistant (R) HER2+ breast cancer-like cell lines. AKT is used as the loading control.
(TIF)

Figure S2 **Schematic representation of components of WP.** Wnt signaling pathway is divided into a ‘canonical’ and a ‘non-canonical’ branch, which are activated following the binding of Wnt ligands to Frizzled trans-membrane receptors. Canonical Wnt signaling causes the activation of beta-catenin–TCF complexes, whereas non-canonical Wnt signal transduction uses a multitude of different downstream effectors instead. In the absence of ligand-induced Wnt activation, newly synthesized beta-catenin is controlled through proteasomal degradation by the ‘destruction’ complex, (Axin, APC, casein kinase 1 and GSK-3β). Binding of Wnt to Frizzled triggers the recruitment of Dishevelled (DVL) and Axin by Frizzled and the Wnt co-receptor LRP, respectively, releasing GSK-3β from the “scaffolding” complex. As a result, unphosphorylated beta-catenin accumulates in the nucleus and interacts with members of the TCF and LEF family of transcription factors to induce transcription of downstream target genes. In drosophila, non-canonical Wnt signaling is required for the establishment of planar cell polarity (PCP), a pathway similar to that controls polarized cell migration during vertebrate development. Downstream effectors of the PCP pathway include small Rho-like GTPases and JNK kinases.
(TIF)

Figure S3 **Characteristic of different TN breast cancer cell lines used in the study.** List of different TN breast cancer cell lines used in the study and their Clinical Subtype, Source^2^, Tumor Type^2^, Gene Cluster^2^, *p53* Levels/Mutational status^2^ /*p53* (IS)^4^/TP53 Amino Acid Mutations^13^, PI3kinaseCA Mutation/PTEN Protein/*pten* Mutation^13^/*Ras* (KRAS, HRAS) Mutation, *BRCA1* Mutation Status, and Epithelial/Mesenchymal Phenotypes.
(TIF)

Figure S4 **Heatmap of differential expression of mRNAs in patients with TN breast tumors.** Hierarchical clustering of differentially expressed mRNAs in TN tumors is compared to luminal and HER2+ breast tumors (Montreal cohort) (16). Tumor biopsies are represented by columns and color labeled according to the breast cancer subtype (blue - TN, gray - HR+, burgundy - HER2+). Differentially expressed mRNAs are represented by rows and those that map to genes that canonically promote Wnt signaling are marked in yellow, those that inhibit Wnt signaling are marked in black.
(TIF)

Figure S5 **Expression of Wnt transcriptional targets in the TN breast cancer cell lines.** Expression of Wnt transcriptional targets in the TN breast cancer cell lines HCC70 (black), MDA-MB-468 (dark grey), HCC38 (light grey), and the luminal-like cell line MCF7 (white) are presented (upper left corner). Error bars represent one standard error of the mean and asterisks (*) indicate p-values < 0.05 determined using a t-test for unequal sample sizes and means. Bar diagram (upper right corner) shows the expression of AXIN1 and AXIN2 transcripts in HCC70 (black), MDA-MB-468 (dark grey), HCC38 (light grey), and MCF7 (white) cell lines. Error bars represent one standard error of the mean and p-values are determined using a t-test for unequal sample sizes and means. Bar diagram of the lower left corner shows the expression of Wnt ligands in the cell lines. Expression of different Wnt signaling components (ligands, receptors, and Wnt transducers) in the TN cell lines HCC70 (black), MDA-MB-468 (dark grey), HCC38 (light grey), and the HR+ cell line MCF7 (white) are presented. Error bars represent one standard error of the mean and asterisks (*) indicate p-values < 0.05 determined using a t-test for unequal sample sizes and means.
(TIF)

Figure S6 **Expression of different components of WP.** Immunoblot (upper panel) shows expression of different components of WP, including, DVL, Axin, and TCF4 in different BT cell lines. Immunoblot (lower panel) shows expression of different components and transcriptional targets of WP in different BT cell lines (H, L and T represents HER2+, luminal and triple negative-like breast cancer cell lines, respectively).
(TIF)
