# Supplementary figures and images for: Differential Activation of Wnt-β-Catenin Pathway in Triple Negative Breast Cancer Increases MMP7 in a PTEN Dependent Manner
Source: PLoS One. 2013 Oct 15;8(10):e77425. doi: 10.1371/journal.pone.0077425 (PMC3797090; doi:10.1371/journal.pone.0077425)

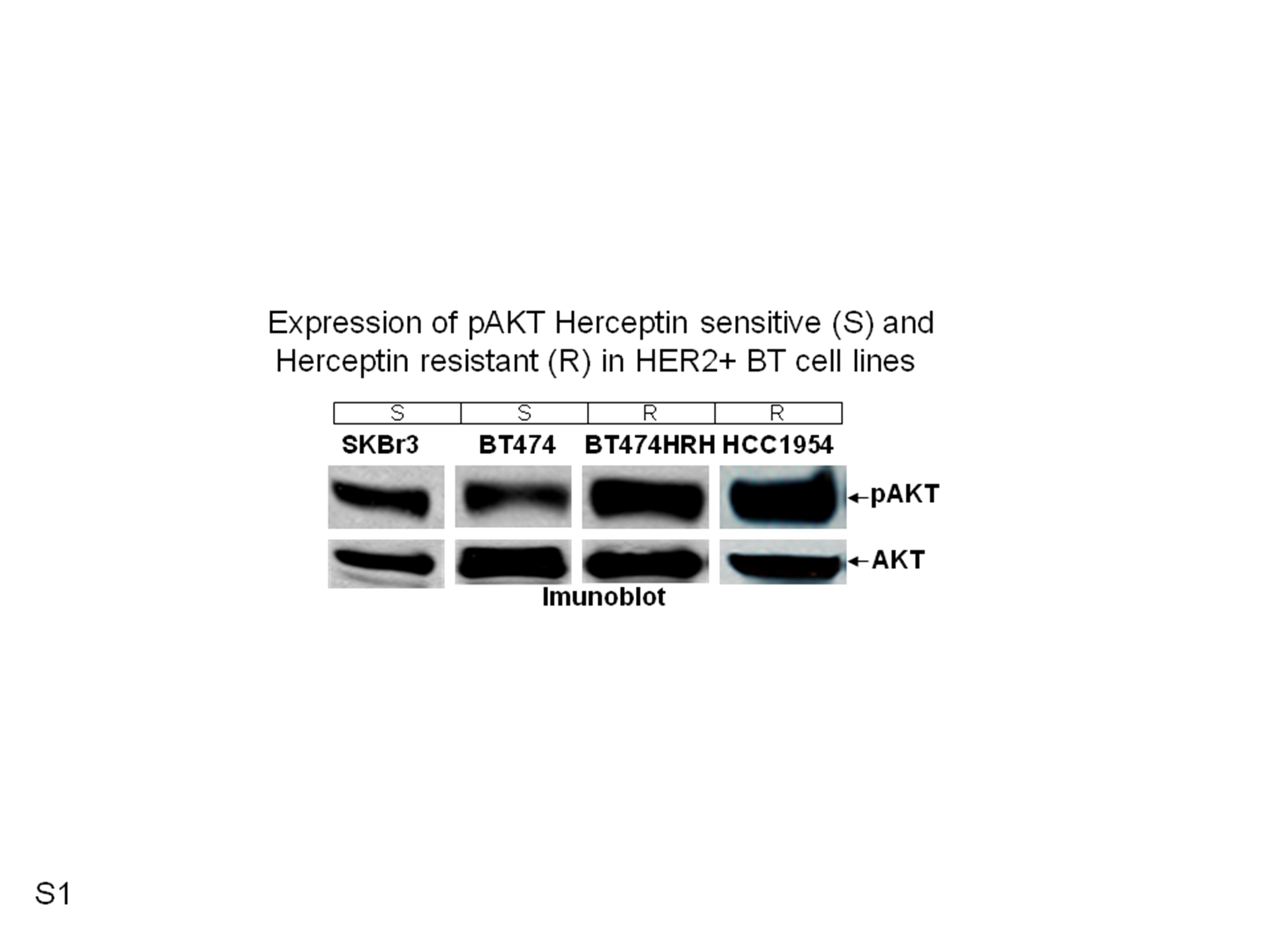

Supplement: Figure S1 — Expression of the levels of pAKT in trastuzumab sensitive (S) and resistant (R) HER2+ breast cancer-like cell lines. Levels of pAKT were determined by immunoblot from two (SKBr3 and BT474) trastuzumab sensitive (S) and two (BT474HR and HCC1954) resistant (R) HER2+ breast cancer-like cell lines. AKT is used as the loading control. (TIF) [file pone.0077425.s001.tif]

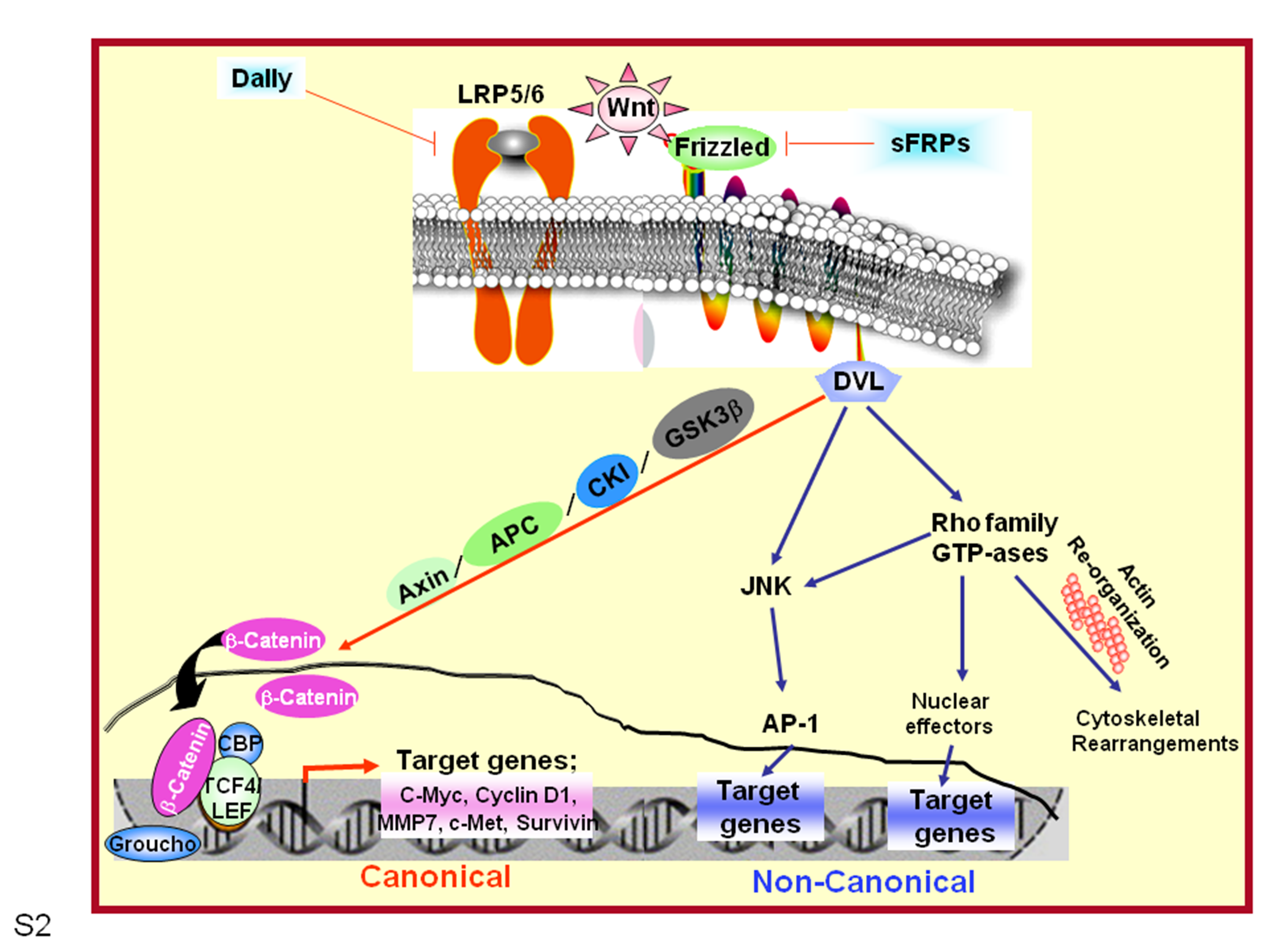

Supplement: Figure S2 — Schematic representation of components of WP. Wnt signaling pathway is divided into a ‘canonical’ and a ‘non-canonical’ branch, which are activated following the binding of Wnt ligands to Frizzled trans-membrane receptors. Canonical Wnt signaling causes the activation of beta-catenin–TCF complexes, whereas non-canonical Wnt signal transduction uses a multitude of different downstream effectors instead. In the absence of ligand-induced Wnt activation, newly synthesized beta-catenin is controlled through proteasomal degradation by the ‘destruction’ complex, (Axin, APC, casein kinase 1 and GSK-3β). Binding of Wnt to Frizzled triggers the recruitment of Dishevelled (DVL) and Axin by Frizzled and the Wnt co-receptor LRP, respectively, releasing GSK-3β from the “scaffolding” complex. As a result, unphosphorylated beta-catenin accumulates in the nucleus and interacts with members of the TCF and LEF family of transcription factors to induce transcription of downstream target genes. In drosophila, non-canonical Wnt signaling is required for the establishment of planar cell polarity (PCP), a pathway similar to that controls polarized cell migration during vertebrate development. Downstream effectors of the PCP pathway include small Rho-like GTPases and JNK kinases. (TIF) [file pone.0077425.s002.tif]

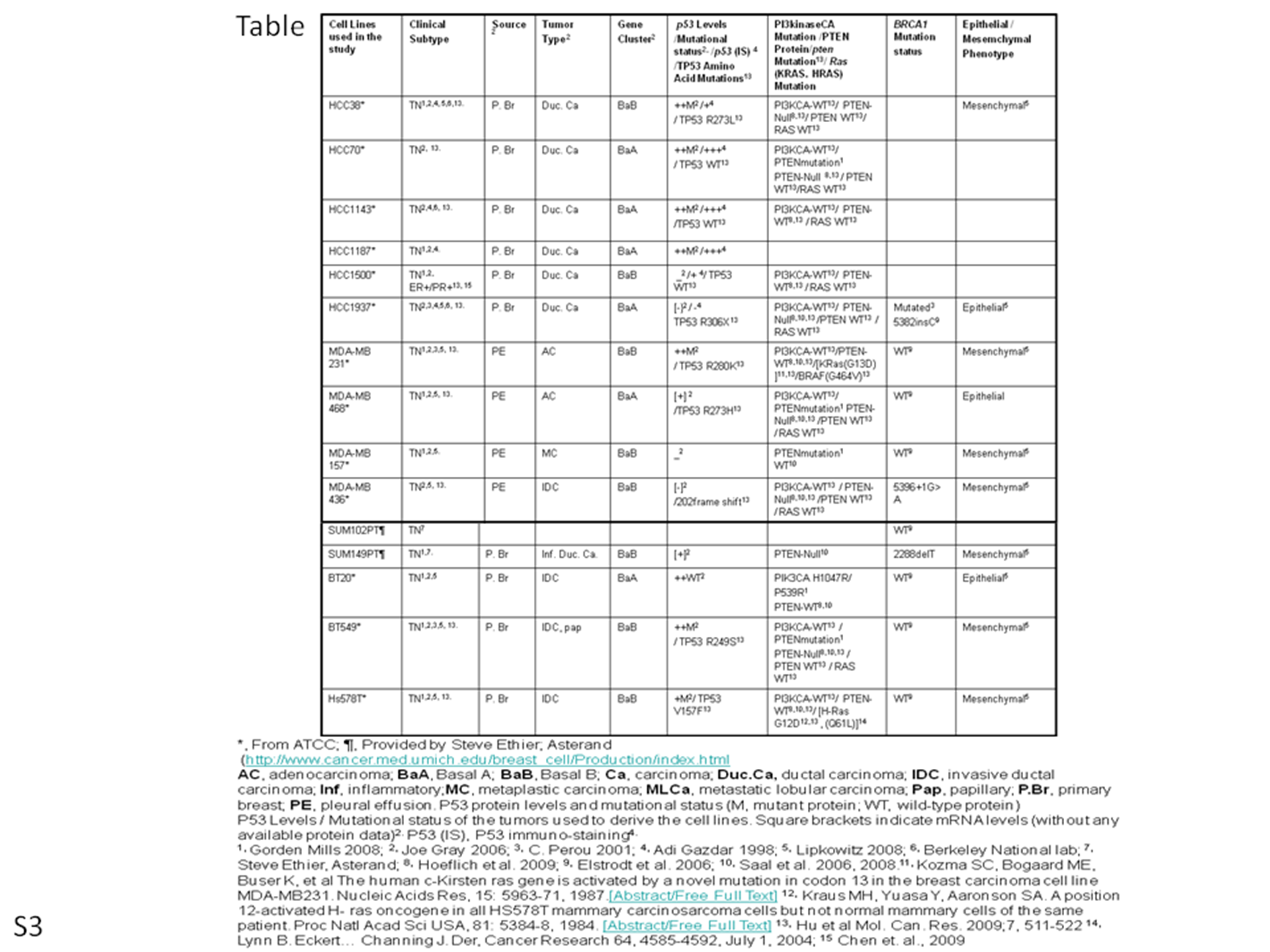

Supplement: Figure S3 — Characteristic of different TN breast cancer cell lines used in the study. List of different TN breast cancer cell lines used in the study and their Clinical Subtype, Source2, Tumor Type2, Gene Cluster2, p53 Levels/Mutational status2 /p53 (IS)4/TP53 Amino Acid Mutations13, PI3kinaseCA Mutation/PTEN Protein/pten Mutation13/Ras (KRAS, HRAS) Mutation, BRCA1 Mutation Status, and Epithelial/Mesenchymal Phenotypes. (TIF) [file pone.0077425.s003.tif]

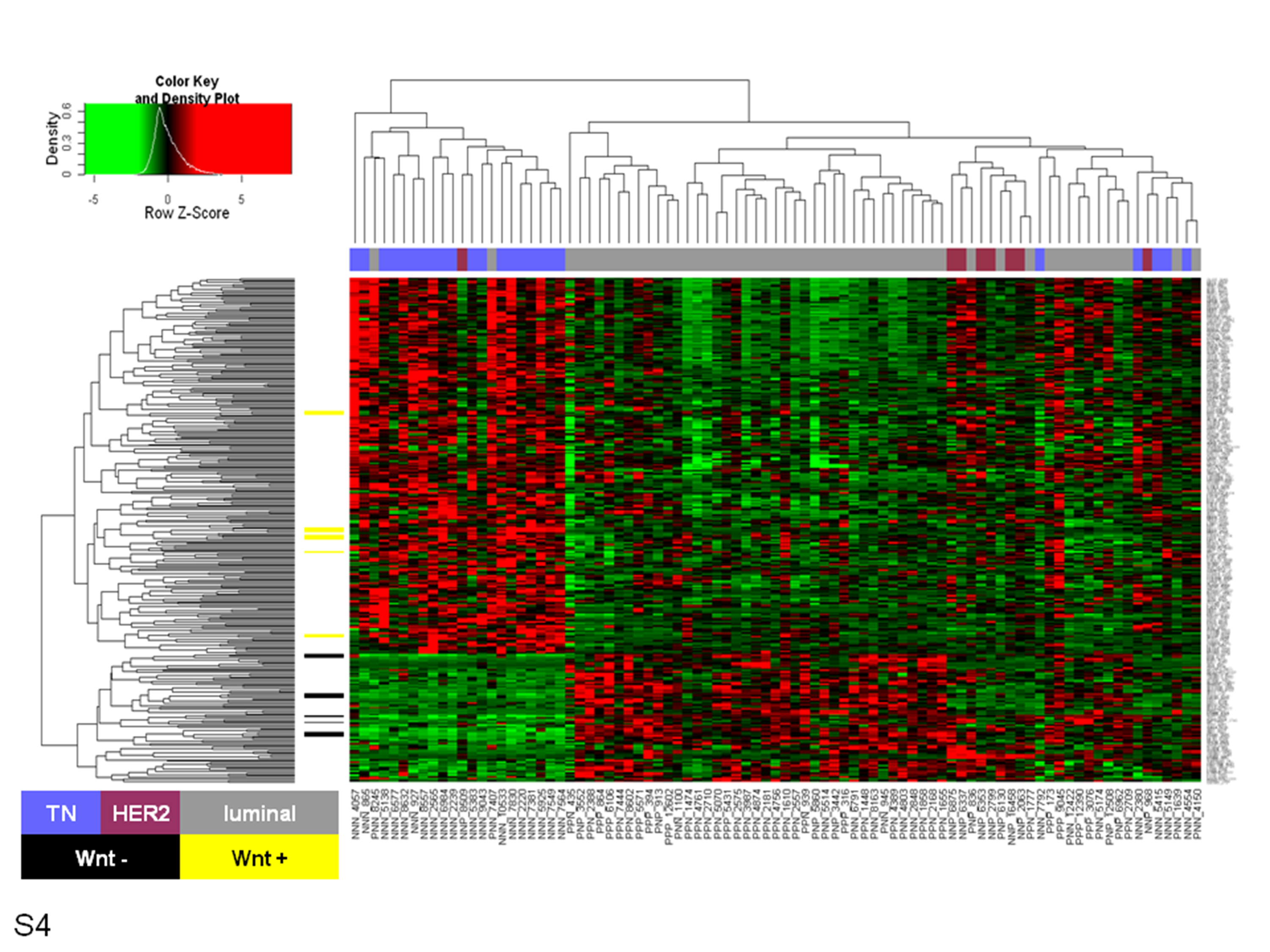

Supplement: Figure S4 — Heatmap of differential expression of mRNAs in patients with TN breast tumors. Hierarchical clustering of differentially expressed mRNAs in TN tumors is compared to luminal and HER2+ breast tumors (Montreal cohort) (16). Tumor biopsies are represented by columns and color labeled according to the breast cancer subtype (blue - TN, gray - HR+, burgundy - HER2+). Differentially expressed mRNAs are represented by rows and those that map to genes that canonically promote Wnt signaling are marked in yellow, those that inhibit Wnt signaling are marked in black. (TIF) [file pone.0077425.s004.tif]

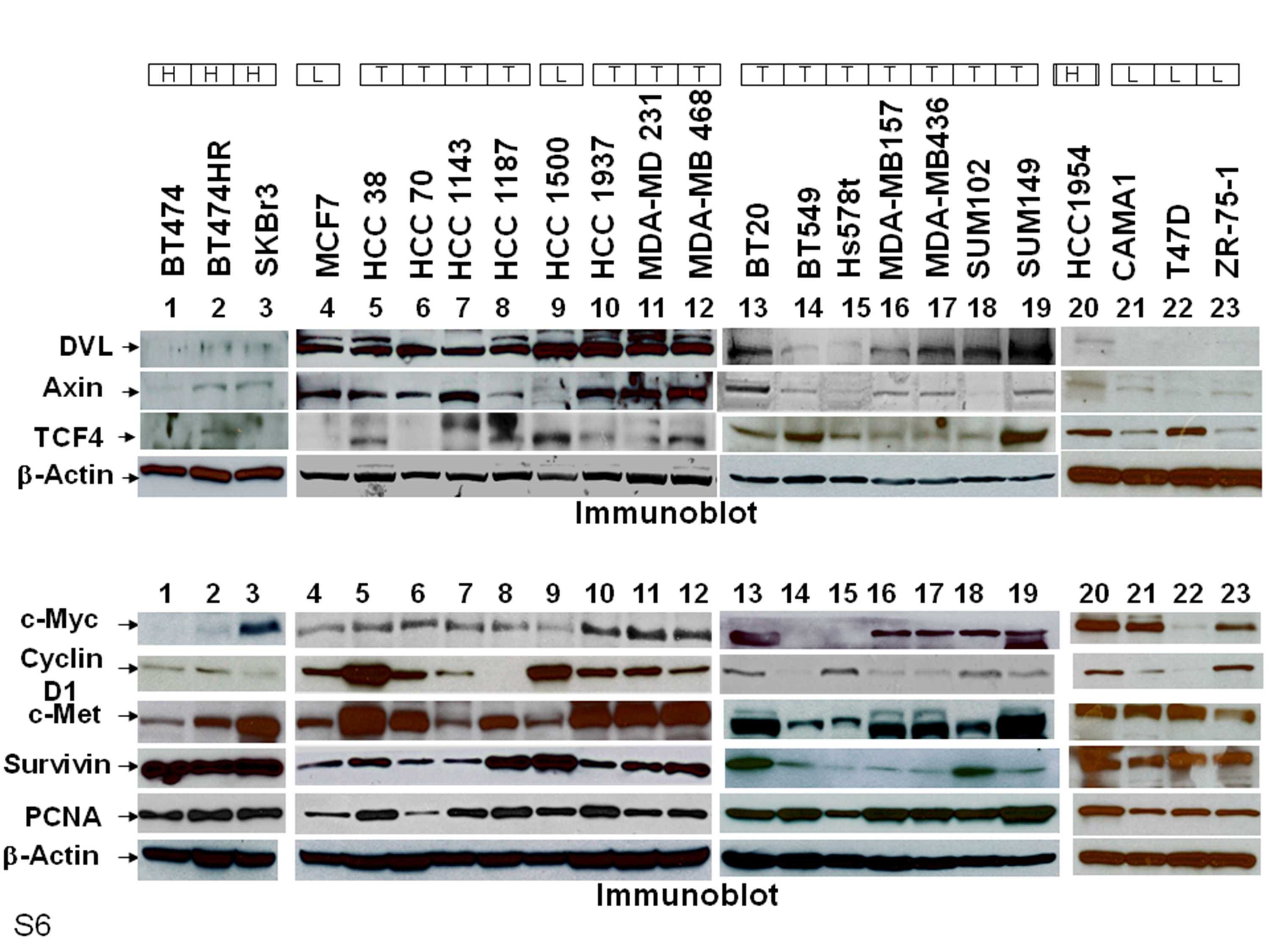

Supplement: Figure S6 — Expression of different components of WP. Immunoblot (upper panel) shows expression of different components of WP, including, DVL, Axin, and TCF4 in different BT cell lines. Immunoblot (lower panel) shows expression of different components and transcriptional targets of WP in different BT cell lines (H, L and T represents HER2+, luminal and triple negative-like breast cancer cell lines, respectively). (TIF) [file pone.0077425.s006.tif]
